# The efficacy and functional consequences of interactions between human spermatozoa and seminal fluid extracellular vesicles

**DOI:** 10.1530/RAF-23-0088

**Published:** 2024-10-04

**Authors:** Cottrell T Tamessar, Amanda L Anderson, Elizabeth G Bromfield, Natalie A Trigg, Shanmathi Parameswaran, Simone J Stanger, Judith Weidenhofer, Hui-Ming Zhang, Sarah A Robertson, David J Sharkey, Brett Nixon, John E Schjenken

**Affiliations:** 1 School of Environmental and Life Sciences, College of Engineering, Science and Environment, The University of Newcastle, University Drive, Callaghan, New South Wales, Australia; 2 Infertility and Reproduction Research Program, Hunter Medical Research Institute, New Lambton Heights, New South Wales, Australia; 3 School of BioSciences, Faculty of Science, Bio21 Institute, The University of Melbourne, Parkville, Victoria, Australia; 4 School of Biomedical Sciences and Pharmacy, College of Health, Medicine and Wellbeing, University of Newcastle, Ourimbah, New South Wales, Australia; 5 Precision Medicine Research Program, Hunter Medical Research Institute, New Lambton Heights, New South Wales, Australia; 6 Central Analytical Facility, Research and Innovation Division, The University of Newcastle, University Drive, Callaghan, New South Wales, Australia; 7 The Robinson Research Institute and School of Biomedicine, University of Adelaide, Adelaide, South Australia, Australia

**Keywords:** extracellular vesicles, fertility, seminal fluid, sperm capacitation, sperm motility, spermatozoa

## Abstract

**Abstract:**

Seminal fluid extracellular vesicles (SFEVs) have previously been shown to interact with spermatozoa and influence their fertilisation capacity. Here, we sought to extend these studies by exploring the functional consequences of SFEV interactions with human spermatozoa. SFEVs were isolated from the seminal fluid of normozoospermic donors prior to assessing the kinetics of sperm-SFEV binding *in vitro*, as well as the effects of these interactions on sperm capacitation, acrosomal exocytosis, and motility profile. Biotin-labelled SFEV proteins were transferred primarily to the flagellum of spermatozoa within minutes of co-incubation, although additional foci of SFEV biotinylated proteins also labelled the mid-piece and head domain. Functional analyses of high-quality spermatozoa collected following liquefaction revealed that SFEVs did not influence sperm motility during incubation at pH 5, yet SFEVs induced subtle increases in total and progressive motility in sperm incubated with SFEVs at pH 7. Additional investigation of sperm motility kinematic parameters revealed that SFEVs significantly decreased beat cross frequency and increased distance straight line, linearity, straightness, straight line velocity, and wobble. SFEVs did not influence sperm capacitation status or the ability of sperm to undergo acrosomal exocytosis. Functional assessment of both high- and low-quality spermatozoa collected prior to liquefaction showed limited SFEV influence, with these vesicles inducing only subtle decreases in beat cross frequency in spermatozoa of both groups. These findings raise the prospect that, aside from subtle effects on sperm motility, the encapsulated SFEV cargo may be destined for physiological targets other than the male germline, notably the female reproductive tract.

**Lay Summary:**

A male’s influence over the biological processes of pregnancy extends beyond the provision of sperm. Molecular signals present in the ejaculate can influence the likelihood of pregnancy and healthy pregnancy progression, but the identity and function of these signals remain unclear. In this study, we wanted to understand if nano-sized particles present in the male ejaculate, called seminal fluid extracellular vesicles, can assist sperm in traversing the female reproductive tract to access the egg. To explore this, we isolated seminal fluid extracellular vesicles from human semen and incubated them with sperm. Our data showed that seminal fluid extracellular vesicles act to transfer molecular information to sperm, but this resulted in only subtle changes to the movement of sperm.

**Graphical abstract:**

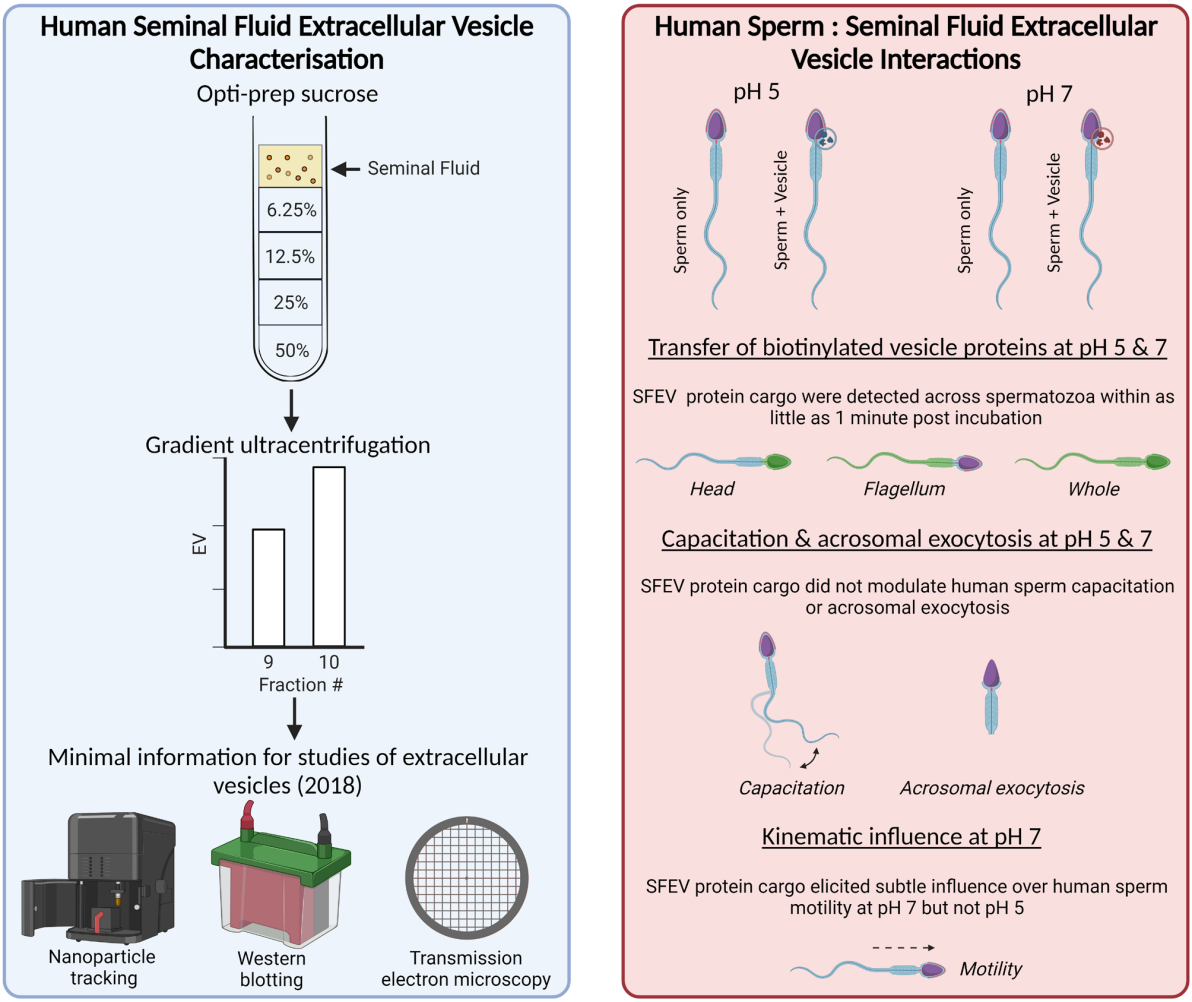

## Introduction

A growing body of evidence suggests that extracellular vesicles (EVs) are important contributors to intercellular communication between somatic tissues (van Niel *et al.* 2018). Indeed, these secreted membrane-bound nanostructures, which typically range in diameter from 50–500 nm, are now recognised as important vectors for relaying macromolecular cargo capable of influencing the phenotype and functional characteristics of a diversity of recipient cells (van Niel *et al.* 2018). This model of intercellular communication appears to hold true in the mammalian reproductive system, wherein EVs have been increasingly implicated in exerting influence over both the male and female reproductive tracts, in addition to the germ cells they accommodate (Simon *et al.* 2018, Trigg *et al.* 2019, Giacomini *et al.* 2020, Tamessar *et al.* 2021). Despite such studies fuelling growing interest in the role of EVs in reproductive success, the full suite of physiological and pathophysiological consequences they exert on reproductive capacity is yet to be resolved.

Since their discovery and initial description in the 1970’s (Ronquist & Hedstrom 1977, Ronquist *et al.* 1978*a*,*b*), seminal fluid EVs (SFEVs) have attracted significant attention as potential regulators of both male and female reproductive function (Arienti *et al.* 1999, Aalberts *et al.* 2014, Rimmer *et al.* 2021, Tamessar *et al.* 2021). Indeed, seminal fluid contains one of the most abundant EV populations of any bodily fluid (Vojtech *et al.* 2014, Mercadal *et al.* 2020, Tamessar *et al.* 2021). SFEVs have been documented to encapsulate a diverse macromolecular cargo, including proteins (Lin *et al.* 2019, Murdica *et al.* 2019*a*, ), nucleic acids (Belleannee *et al.* 2013, Vojtech *et al.* 2014), and lipids (Brouwers *et al.* 2013), a portion of which are thought to influence sperm function as well as exert regulatory control over cells lining the female reproductive tract (Kelly *et al.* 1991, Arienti *et al.* 1999, Carlsson *et al.* 2003, Park *et al.* 2011, Murdica *et al.* 2019*a*,*b*, Rodriguez-Caro *et al.* 2019, Vojtech *et al.* 2019, George *et al.* 2020). While studies have largely attributed the prostate and epididymis as the primary sources of SFEVs, emerging evidence suggests that EVs can be isolated from virtually all tissues of the male reproductive tract, indicating that the composition of this EV population may be far more heterogeneous and complex than previously ascribed (Thimon *et al.* 2008, Belleannee *et al.* 2013, Hoog & Lotvall 2015, Murdica *et al.* 2019*b*). This notion is supported by evidence of variations in the protein and small non-coding RNA composition of SFEV cargo that have been characterised so far, as well as the differential responses this cargo can exert (e.g. on sperm function) when isolated from the ejaculates of vasectomised men as opposed to men with a vasovasostomy procedure (Thimon *et al.* 2008, Belleannee *et al.* 2013).

Since spermatozoa are bathed in seminal fluid from the moment of ejaculation, these cells have drawn attention as one of the key potential targets for SFEV cargo. In this context, previous studies have demonstrated that SFEVs can indeed interact with spermatozoa in an *in vitro* setting (Arienti *et al.* 1997, Carlini *et al.* 1997, Palmerini *et al.* 1999, Park *et al.* 2011, Wang *et al.* 2022), although the functional consequences of such interactions remain contentious. Thus, some studies report that SFEVs are capable of improving sperm motility and stimulating capacitation and acrosomal exocytosis (Fabiani *et al.* 1994, Palmerini *et al.* 1999, Palmerini *et al.* 2003, Park *et al.* 2011, Murdica *et al.* 2019*b*, Wang *et al.* 2022). Extending these observations, other studies have shown that SFEVs offer protection to spermatozoa against the acidic environment they encounter following deposition into the female reproductive tract (Arienti *et al.* 1999). Such functions may be conserved across mammalian species, with SFEVs demonstrated to influence sperm function in diverse species including pig (Siciliano *et al.* 2008, Piehl *et al.* 2013, Du *et al.* 2016), mouse (Park *et al.* 2011), cattle (Kumar *et al.* 2019), stallion (Aalberts *et al.* 2013), dog (Mogielnicka-Brzozowska *et al.* 2015, Zelli *et al.* 2017), and rooster (Cordeiro *et al.* 2021). Despite this evidence, inconsistencies in the methodology used for SFEV isolation have contributed to alternative interpretations of the functional consequences being attributed to sperm–SFEV interactions. For example, human studies have linked SFEVs to inhibition of sperm function, including suppression of premature capacitation and acrosomal exocytosis (Bechoua *et al.* 2011, Pons-Rejraji *et al.* 2011). Other studies allude to the prospect that SFEVs only regulate sperm function indirectly via signalling mediators, such as progesterone and/or pH changes associated with passage through the differing microenvironments of the female reproductive tract (Park *et al.* 2011).

To address these controversies, in this study we isolated SFEVs from human seminal fluid following the conventions specified by the International Society for Extracellular Vesicles (Thery *et al.* 2018) and then systematically explored the nature and functional consequences of human sperm-SFEV interactions using a readily tractable *in vitro* co-culture system that we previously optimised for this purpose in mice (Zhou *et al.* 2019, Trigg *et al.* 2021). Specifically, our exploration of the functional consequences of human sperm-SFEV interactions utilised computer-assisted sperm analysis (CASA) and immunocytochemical assays to determine influences on sperm motility parameters, capacitation status, and capacity to undergo an acrosome reaction.

## Materials and methods

### Ethics statement

The experiments described in this study were conducted using human semen samples sourced from the University of Newcastle’s Reproductive Science Group sperm donor program and were performed in accordance with procedures approved by the University of Newcastle’s Human Research and Ethics Committee (approval no. H-2013-0319). All donors provided informed written consent, and samples were de-identified prior to delivery to the laboratory except for the assignment of a unique identification number. Prior to use in experiments, all semen samples were confirmed as normozoospermic as defined by the criteria set forth by the World Health Organisation (World Health Organisation 2010).

### Chemicals and reagents

All reagents were purchased from Merck and were of molecular biology or research quality unless otherwise specified.

### Preparation of human spermatozoa

Semen samples were produced by masturbation following sexual abstinence of at least 48 h and stored at 37°C prior to processing. Samples were either processed following completion of liquefaction within 1 h of ejaculation or, as required, within 15 min of ejaculation prior to liquefaction. Semen samples were fractionated over a discontinuous Percoll density gradient (40% v/v and 80% v/v Percoll) by centrifugation at 500 ***g*** for 30 min as described previously (Aitken *et al.* 1993, Nixon *et al.* 2011). After centrifugation, high-quality populations of spermatozoa were retrieved from the pellet formed at the bottom of the tube (Aitken *et al.* 1993). In some experiments, low-quality spermatozoa were collected from the interface of the 40% and 80% Percoll gradient. Spermatozoa from both groups were resuspended in modified Biggers, Whitten, and Whittingham medium (BWW) (91.5 mM NaCl, 4.6 mM KCl, 1.7 mM CaCl_2_·_2_H_2_O, 1.2 mM KH_2_PO_4_, 1.2 mM MgSO_4_·7H_2_O, 25 mM NaHCO_3_, 5.6 mM D-glucose, 0.27 mM sodium pyruvate, 44 mM sodium lactate, 5 U/mL penicillin, 5 mg/mL streptomycin, 20 mM HEPES buffer, and 1 mg/mL polyvinyl alcohol (PVA); osmolarity of 290–319 mOsm/kg) (Bromfield *et al.* 2015) and washed by centrifugation at 500 ***g*** for 15 min before resuspension in BWW in preparation for experimental analyses. Acellular seminal fluid was recovered from the top layer of the density gradient and prepared for use as described below.

### Seminal fluid extracellular vesicle isolation and characterisation

Seminal fluid was retained following sperm enrichment as a clearly defined layer at the top of the Percoll density gradient. These samples were then sequentially centrifuged with increasing velocity at 4°C to eliminate contaminating cells and cellular debris (500 ***g***, 5 min; 2,000 ***g***, 5 min; 2,000 ***g***, 5 min; 8000 ***g***, 5 min; 17,000 ***g***, 20 min; and repeat centrifugations at 17,000 ***g*** for 10 min until a pellet was no longer visible). The resultant supernatant was layered onto a discontinuous density gradient modified from previous studies (comprising 50%, 25%, 12.5%, and 6.25% OptiPrep suspension diluted in a solution of 0.25 M sucrose, 10 mM Tris HCl, pH 7.5) (Reilly *et al.* 2016, Zhou *et al.* 2019) and centrifuged at 158,300 ***g*** for 18 h at 4°C. After centrifugation, 12 equivalent fractions (185 µL per fraction) were collected and diluted in 3 mL phosphate-buffered saline (PBS; 137 mM NaCl, 2.7 mM KCl, 8 mM Na_2_HPO_4_, and 2 mM KH_2_PO_4_, pH 7.4). Each fraction was then centrifuged at 100,000 ***g*** for 3 h at 4°C to remove contaminating soluble fluid plasma proteins that partitioned to the same density fraction as the EVs. Pellets were solubilised in the relevant buffers as required for downstream application. Each of the 12 fractions was characterised in accordance with the MISEV2018 guidelines for EV research (Thery *et al.* 2018), as described below, to identify SFEV-containing fractions.

### Sodium dodecyl sulphate-polyacrylamide gel electrophoresis (SDS-PAGE), silver staining, and immunoblotting

Proteins partitioning into each of the 12 collected fractions were solubilised in SDS-PAGE extraction buffer (2% SDS, 0.3 M sucrose, 0.1875 M Tris-HCl (pH 6.8) containing protease inhibitors (Roche Holding AG, Basel, Switzerland), and boiled at 100°C for 5 min. Insoluble material was pelleted by centrifugation at 21,000 ***g*** for 10 min at 4°C, and the concentration of protein present in the resultant supernatant was quantified using a Detergent Compatible (DC) protein assay kit (Bio-Rad Laboratories).

To assess the profile of proteins present in each of the 12 fractions, an equivalent volume of each fraction was diluted into SDS-PAGE loading buffer (2% v/v mercaptoethanol, 2% w/v SDS, and 10% w/v sucrose in 0.1875 M Tris, pH 6.8, with bromophenol blue) and resolved by SDS-PAGE (constant voltage of 150 V for 45 minutes) using Mini-Protean TGX Gels (4-20%) (Bio-Rad Laboratories). Gels were then either prepared for silver staining or transferred to nitrocellulose membranes using standard procedures (Zhou *et al.* 2017). Please see Supplementary Table S1 (see section on supplementary materials given at the end of this article) for a complete list of all antibodies used in this study, including the concentrations at which they were used. For immunoblotting, an equivalent volume of all 12 fractions was initially probed for the presence of flotillin 1 (using anti-FLOT1 antibodies) to identify SFEV-enriched fractions. Analysis of concentrated EV suspensions from fractions 9 and 10 then took place using a volume of SFEV or seminal fluid control protein equivalent to 3 µL of starting seminal fluid material for additional positive (anti-CD63 antibodies) and negative apolipoprotein A1 (anti-APOA1 antibodies) EV markers. Membranes were initially blocked using either 3% BSA in Tris-buffered saline (20 mM Tris, 150 mM NaCl, pH 7.6) with 0.1% (v/v) Tween 20 (TBST) (anti-FLOT1 antibodies), 5% BSA in TBST (anti-APOA1 antibodies) or 5% skim milk in TBST (anti-CD63 antibodies) for 1 h at room temperature. They were then incubated with primary antibodies prepared in either 1% BSA in TBST (anti-FLOT1 antibody), 5% BSA in TBST (anti-APOA1 antibody) or 1% skim milk in TBST (anti-CD63 antibody) overnight with gentle rotation at 4°C. Blots were subsequently washed with three changes of TBST, followed by incubation with horseradish peroxidase (HRP)-conjugated goat anti-rabbit IgG secondary antibodies (Thermo Fisher Scientific) at room temperature for 1 h. After three additional washes in TBST, labelled proteins were detected using an enhanced chemiluminescence kit (GE Healthcare). Immunoblots and silver-stained gels were imaged using an ImageQuant LAS-4000 Biomolecular Imager (GE Healthcare).

### Assessment of the physical characteristics of seminal fluid extracellular vesicles

Nanoparticle tracking analysis (NTA) was used to determine particle size distribution and concentration of SFEV samples as previously described (Brzozowski *et al.* 2018). Briefly, concentrated EV suspensions from fractions 9 and 10 were diluted 1:1000 in 0.1 µM filtered PBS before introduction into the chamber of a Nanosight NS300 (Malvern Panalytical, Malvern, UK) using a syringe pump (Harvard Apparatus, ATA Scientific) at a speed setting of 40. The NS300 was adjusted so particles were in the focal plane and a scientific CMOS camera captured 3 × 60-second videos per sample. Settings were kept constant between samples. Particle size distribution and concentration were determined using NTA Software Suite Version 3. Alternatively, concentrated EV suspensions were fixed and processed for transmission electron microscopy (TEM) using methodology modified from (Polanco *et al.* 2016). Prior to analysis, fractions 9 and 10 were combined and fixed in 100 µL of 2% (v/v) glutaraldehyde in filtered PBS. Following 3 h of centrifugation at 100,000 ***g*** at 4°C, SFEVs were resuspended in a final volume of 100 µL filtered PBS. A 10 µL aliquot of this suspension was applied to a silicon nitride TEM grid (ProSciTech, Kirwan, QLD, Australia), to which 5 µL of specimen solution was added onto the surface prior to placing the grid in a Laurell H6-23 spin coater (Laurell Technologies, Lansdale, PA, USA) and incubating at a rotational speed of 4000 r.p.m. for 30 seconds. SFEVs were then sequentially stained in 2% (w/v) methanolic uranyl acetate for 15 min and Reynold’s lead citrate solution for 10 min at room temperature, before being imaged using a JEOL JEM-1200EXII Transmission Electron Microscope (JEOL Ltd, Tokyo, Japan) under 80 KV accelerating voltage.

### Co-incubation of human SFEVs with spermatozoa

Isolated SFEVs were resuspended in 500 µL PBS and subjected to biotin labelling by reaction with either membrane-impermeable (EZ-Link sulfo-NHS-LC-Biotin, Thermo Fisher Scientific) or membrane-permeable (EZ-Link BMCC-Biotin, Thermo Fisher Scientific) biotin reagents at a concentration of 50 µg biotin/1 mg SFEV protein in accordance with the manufacturer’s instructions. Labelling reactions were conducted at room temperature for 30 min, then stored at 4°C in the dark overnight with constant rotation. Unbound biotin reagent was subsequently quenched by diluting the SFEV suspensions in 50 mM glycine in PBS prior to centrifugation at 100,000 ***g*** for 3 h at 4°C. The resultant SFEV pellets were resuspended in non-capacitating BWW (i.e. BWW prepared without NaHCO_3_ and buffered to a pH of either 5 or 7) prior to co-incubation with spermatozoa (from a different cohort of donors) at a ratio of 10 million sperm cells per 800 µg of SFEV protein. Sperm-SFEV co-incubations were conducted in an atmosphere of 5% CO_2_ at 37°C, on a rotating platform for a maximum of either 60 min (EZ-Link sulfo-NHS-LC-Biotin) or 180 min (EZ-Link BMCC-Biotin), with a portion of each sample recovered at regular intervals over the course of the incubation. At the completion of co-incubation, spermatozoa were washed three times by gentle centrifugation (500 ***g***, 3 min) in appropriate media and fixed in 4% paraformaldehyde (PFA) under darkened conditions at room temperature for 15 min. Controls for these experiments included a separate population of spermatozoa labelled directly with both biotin reagents in the absence of SFEVs, spermatozoa incubated with unlabelled SFEVs (to control for the non-specific biotinylation of sperm proteins and the presence of endogenous biotin, respectively), and spermatozoa incubated in media containing quenched biotin to confirm the efficacy of quenching of unreacted biotin.

Fixed samples were settled on poly-L-lysine coverslips overnight at 4°C. Cells were blocked with 3% (w/v) BSA in PBS at room temperature prior to washing in PBS and incubation with AlexaFluor 488 conjugated streptavidin (0.25 µg; Thermo Fisher Scientific) diluted in 1% (w/v) BSA/PBS at 37°C for 1 h. Coverslips were washed three times in PBS and counterstained with 4′,6-diamidino-2-phenylindole (DAPI; 2 μg/mL) at room temperature for 30 seconds before mounting in antifade solution (10% Mowiol 4–88 with 30% glycerol in 0.2 M Tris (pH 8.5) and 2.5% 1,4-diazabicyclo-(2.2.2)-octane (DABCO)). Coverslips were visualised using fluorescence microscopy (Zeiss Axio Imager A2; Carl Zeiss AG, Jena, Germany), and SFEV cargo transfer (as determined by the presence of biotin labelling) and cargo localisation was assessed for a minimum of 100 spermatozoa per sample.

### Assessment of the impact of seminal fluid extracellular vesicles on sperm motility parameters

Populations of SFEVs were resuspended in BWW (either pH 5 or 7) and co-incubated with spermatozoa at a ratio of 10 million sperm per 800 µg of SFEV protein in an atmosphere of 5% CO_2_ at 37°C with gentle rotation over an incubation period of 300 min. At regular intervals throughout the incubation (i.e. 1, 5, 15, 30, 60, 180, and 300 min), a sperm sample was recovered, and their motility parameters were objectively evaluated using a Hamilton Thorne IVOS II Clinical CASA System (Version 12; Hamilton Thorne Biosciences, MA, USA). Motility was assessed by scoring 200 cells from each sample using standard settings for human spermatozoa (30 frames acquired at a frame rate of 60 Hz in 20 µm deep chambers at a constant temperature of 37°C) in a standard four-chamber slide (Leja Products B.V., Nieuw-Vennep, Netherlands).

### Assessment of the impact of SFEVs on sperm capacitation and acrosomal exocytosis

Populations of spermatozoa co-incubated with SFEVs were fixed in 4% PFA for assessment of the impact of SFEVs on capacitation (i.e. immunostaining for phosphotyrosine) and acrosomal status (i.e. affinity labelling with fluorescein isothiocyanate-conjugated peanut agglutinin (FITC-PNA)) as described below. Positive controls included sperm induced to capacitate via incubation in BWW supplemented with 3 mM pentoxifylline and 5 mM dibutyryl cyclic adenosine monophosphate (termed ‘capacitation media’ throughout), and sperm incubated in 2.5 μM A23187 to induce acrosomal exocytosis (Mitchell *et al.* 2007, Mitchell *et al.* 2008, Redgrove *et al.* 2011). Negative controls included sperm held in a non-capacitated state via incubation in BWW medium prepared in the absence of bicarbonate as previously described (Redgrove *et al.* 2011). After incubation, sperm cells were settled on poly-l-lysine coverslips overnight at 4°C and prepared for immunostaining via permeabilisation in 0.2% Triton X-100 for 15 min and subsequent blocking in 3% (w/v) BSA/PBS for 1 h. Cells were then incubated with anti-phosphotyrosine (pTyr) mouse monoclonal IgG1 antibodies (8 µg/mL) at 37°C for 1.5 h. Coverslips were washed three times in PBS and incubated in goat anti-mouse IgG antibodies conjugated to Alexa Fluor 488 (5 µg/mL) (Thermo Fisher Scientific) at 37°C 1 h. Following three washes in PBS, cells were incubated with FITC-PNA (1 μg/μL) (Thermo Fisher Scientific) at 37°C for 25 minutes. Coverslips were then washed in PBS and counterstained in DAPI at room temperature for 2 min. Stained coverslips were rinsed with PBS before being mounted onto glass slides and visualised to assess capacitation and acrosomal status using fluorescence microscopy (Zeiss Axio Imager A2). For each event, a total of 100 spermatozoa were counted in each sample and the corresponding percentage of capacitated (i.e. phosphotyrosine labelling of the entire flagellum) and acrosome-reacted (i.e. no PNA labelling of the acrosomal domain) spermatozoa was determined.

### Statistical analysis

Experiments reported in this study were repeated on at least five biological replicates. For biotinylation experiments, each biological replicate represented pooled samples of spermatozoa and SFEVs isolated from two donors. For sperm functional analyses, each biological replicate represented the spermatozoa and SFEVs isolated from a single donor. Graphical data were prepared using GraphPad Prism version 9.0.0 for Windows (GraphPad Software www.graphpad.com) and are presented as mean values ± s.e.m. Differences between groups attributable to incubation time and/or the presence of SFEVs were assessed using a 2-way ANOVA with Tukey’s multiple comparisons test with *P* < 0.05 defined as statistically significant.

## Results

### Enrichment and physical characterisation of human seminal fluid extracellular vesicles

**Figure 1:**
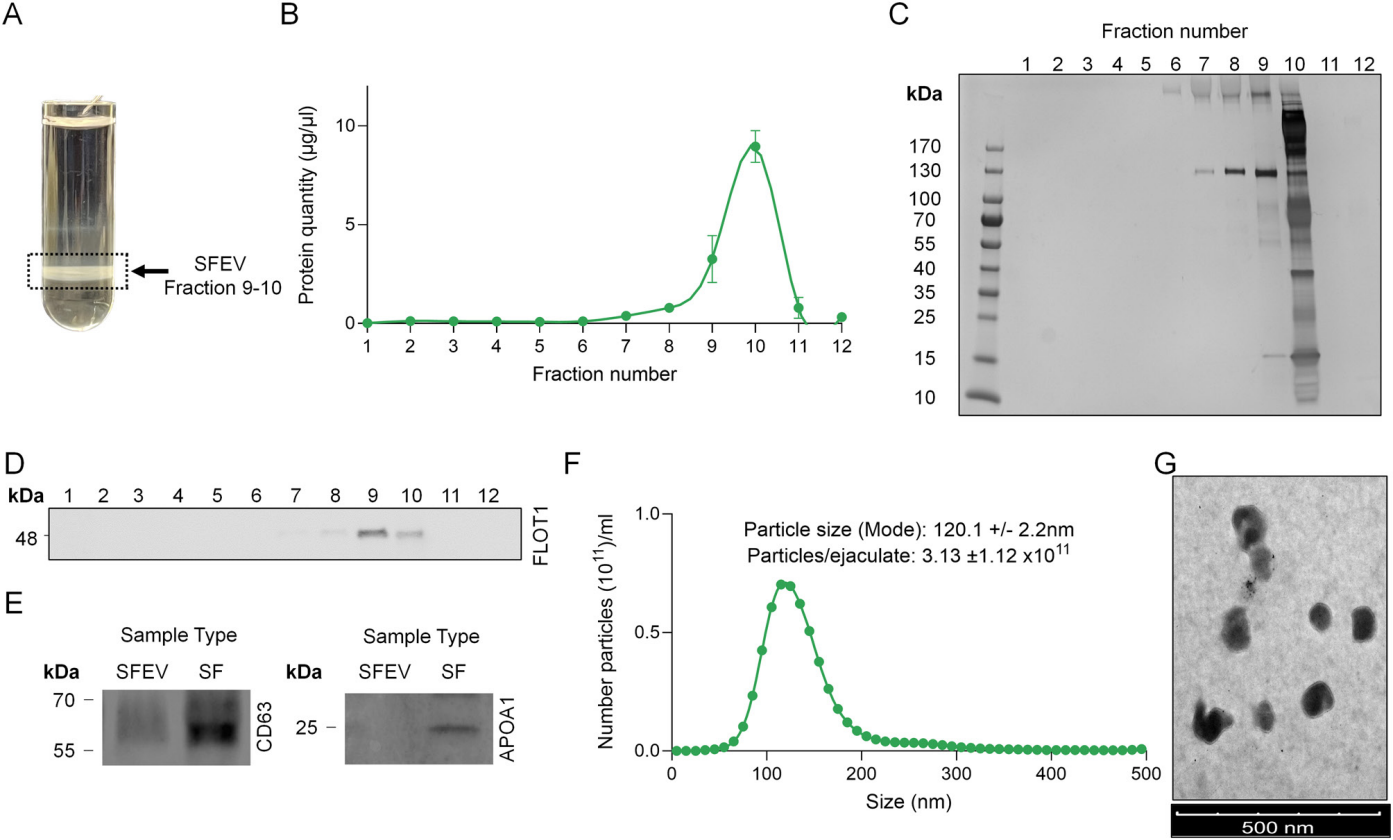
Characterisation of human seminal fluid extracellular vesicles. Seminal fluid extracellular vesicles (SFEVs) were isolated using a modified density gradient ultracentrifugation approach. (A) After centrifugation, an image was taken of the gradient prior to the collection of 12 equivalent fractions. These fractions were subjected to (B) quantitative assessment of total protein content and (C) qualitative assessment of the protein profile. Based on this analysis, and the identification of (D) EV-enriched fractions by immunoblotting all fractions for otillin 1 (FLOT1), fractions 9 and 10 were selected and combined for downstream analysis. This analysis included: (E) Immunoblotting to assess the presence of additional recognised EV markers, CD63 molecule, in addition to the absence of the non-EV protein marker apolipoprotein A1 (APOA1) using seminal fluid protein (SF) as a positive control, (F) determination of particle size and heterogeneity using nanoparticle tracking analysis. Data are represented as particle size (*x*-axis) and particle number (*y*-axis). (G) Transmission electron microscopy to examine the ultrastructural properties of isolated SFEVs (scale bar = 500 µm). All experiments were repeated using *n* = 5 independent biological replicates.

Although seminal fluid is an abundant source of EVs, a major challenge to the assessment of EV function lies in the effective isolation of highly enriched populations of EVs (Thery *et al.* 2018). Accordingly, herein we elected to use a low-recovery, high-specificity approach as recommended in the MISEV 2018 guidelines (Thery *et al.* 2018). This approach involved the optimisation of an OptiPrep gradient density-based centrifugation methodology that we have previously used for the isolation of mouse epididymosomes (Reilly *et al.* 2016, Zhou *et al.* 2019). To establish this method, seminal fluid was collected from normozoospermic donors and separated away from contaminating cellular components of the ejaculate using a standard colloidal silica density gradient centrifugation protocol (Aitken *et al.* 1993, Nixon *et al.* 2011). Following a series of sequential low-speed centrifugation steps, seminal fluid was overlaid onto a discontinuous non-ionic, iodixanol-based density gradient and subjected to ultracentrifugation, resulting in the separation of a visible opalescent layer of high-density material commensurate with the expected density of EVs (Fig. 1A).

Following ultracentrifugation to remove contaminating soluble proteins, quantitative (Fig. 1B) and qualitative (Fig. 1C) analysis of the remaining vesicle-encapsulated proteins present in each density gradient fraction demonstrated that the majority were detected in fractions 9 and 10, indicating SFEV presence in these high-density layers (Fig. 1A–C). Notably, there was no significant variation across multiple donors in the location of this visible opalescent layer. Additionally, immunoblot assessment using the well-characterised EV marker, FLOT1 (Thery *et al.* 2018), confirmed that this protein was abundant in fractions 9 and 10 (Fig. 1D). Owing to the fact that the majority of proteins were detected in fractions 9 and 10 (Fig. 1B and C), and that these fractions contained the highest abundance of the EV marker, FLOT1 (Fig. 1D) these fractions were selected for further characterisation.

Additional analysis of the EV-enriched fractions using seminal fluid protein as a positive control confirmed that pooled fractions 9 and 10 contained the EV marker, CD63 (Fig. 1E). By comparison, the non-EV protein marker APOA1, which was detected in seminal fluid, was not detected in the SFEV-enriched fractions (Fig. 1E). As further confirmation that fractions 9 and 10 were enriched in EVs, particle assessment using NTA and TEM technology platforms revealed the presence of a heterogeneous population of vesicles, the (mode ± s.e.m.) diameter of which was predicted to be ~120.1 ± 2.2 nm, with a particle concentration of 3.1 ± 1.1 × 10^11^ particles/ejaculate (Fig. 1F and G). Ultrastructural analysis confirmed the presence of vesicles within the size range identified by NTA with a double-layer membrane structure (Fig. 1G) as expected for EVs (Hoog & Lotvall 2015, van Niel *et al.* 2018). Accordingly, those fractions with notable enrichment of SFEVs (i.e. fractions 9 and 10) were utilised for the remaining studies reported herein.

### SFEVs transfer biotinylated proteins to human spermatozoa

**Figure 2:**
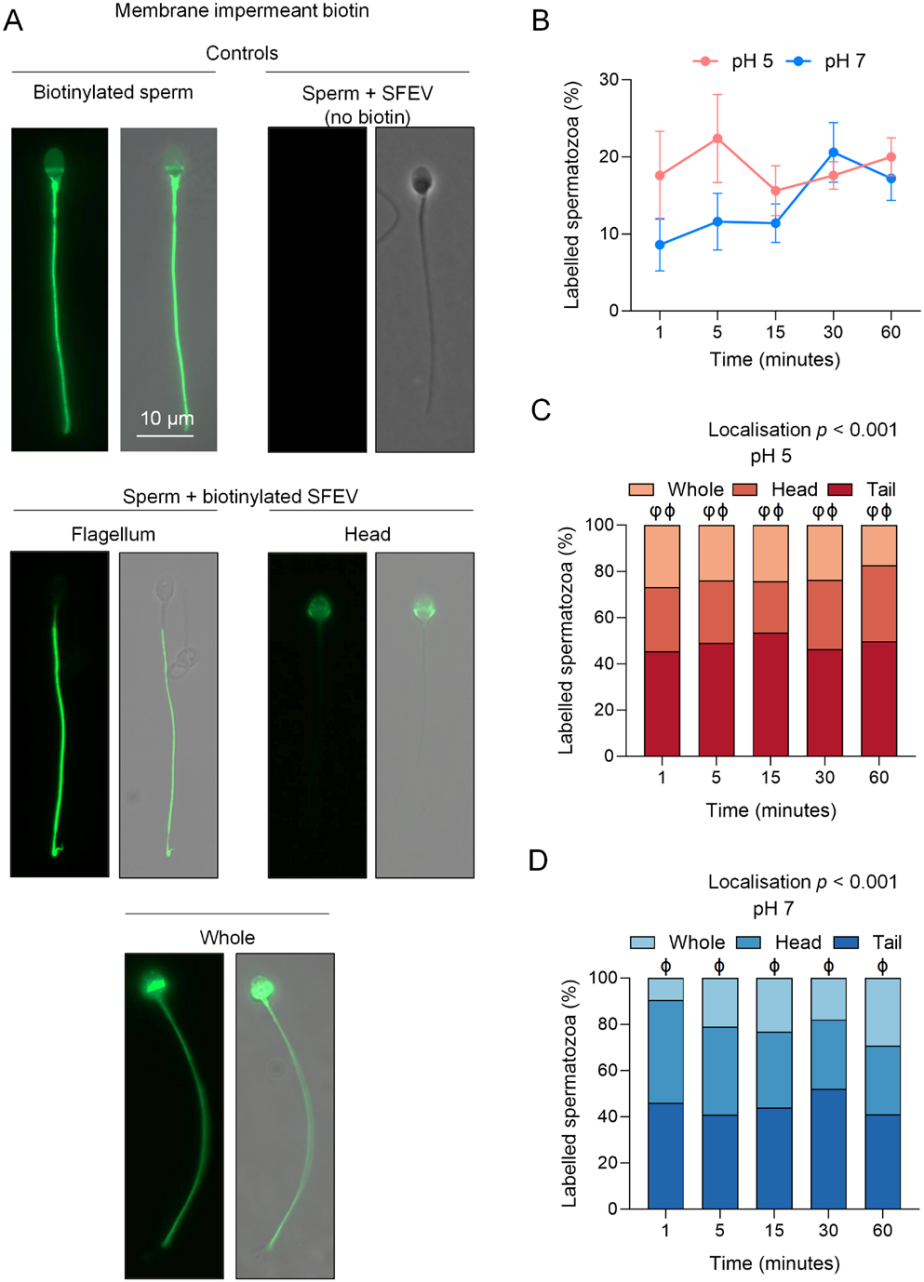
Assessment of seminal fluid extracellular vesicle (SFEV) transfer interaction with human spermatozoa. The localisation of biotinylated SFEV proteins was used as a surrogate to assess the specificity, efficacy, kinetics, and distribution of SFEV interactions with human spermatozoa. For this purpose, isolated SFEVs (i.e. EVs present in the pooled fractions 9 and 10 – see Fig. 1) were labelled with a membrane-impermeable biotin reagent, prior to quenching of the biotinylation reaction and co-incubation with spermatozoa for 60 min (with regular sampling at intervals of 1, 5, 15, 30, and 60 min). Spermatozoa were subsequently affinity-labelled with AlexaFluor 488 conjugated streptavidin. (A) Representative images indicating the localisation patterns obtained following direct biotinylation of spermatozoa (i.e. no SFEVs), sperm incubation with unlabelled SFEVs (i.e. no biotin), and sperm incubation with biotinylated SFEVs (scale bar = 10 µm). (B) The percentage of spermatozoa displaying the uptake of biotinylated proteins and the domains into which these proteins were distributed at (C) pH 5 and (D) pH 7 were determined by examining the fluorescence staining of a minimum of 100 cells per sample. All experiments were repeated using *n* = 5 independent biological replicates, with representative images being shown and graphical data in panels presented as (B) mean ± s.e.m., and (C and D) stacked bar chart showing mean of each group. Data were assessed by 2-way ANOVA with Tukey’s multiple comparisons test (φ, *P* < 0.05 comparing tail vs head labelling, ϕ, *P* < 0.05 comparing tail vs whole cell labelling). Differences in the overall effects of Localisation or Time are presented as text depicting the *P* value in each panel where a significant difference (*P <* 0.05) was detected.

**Figure 3:**
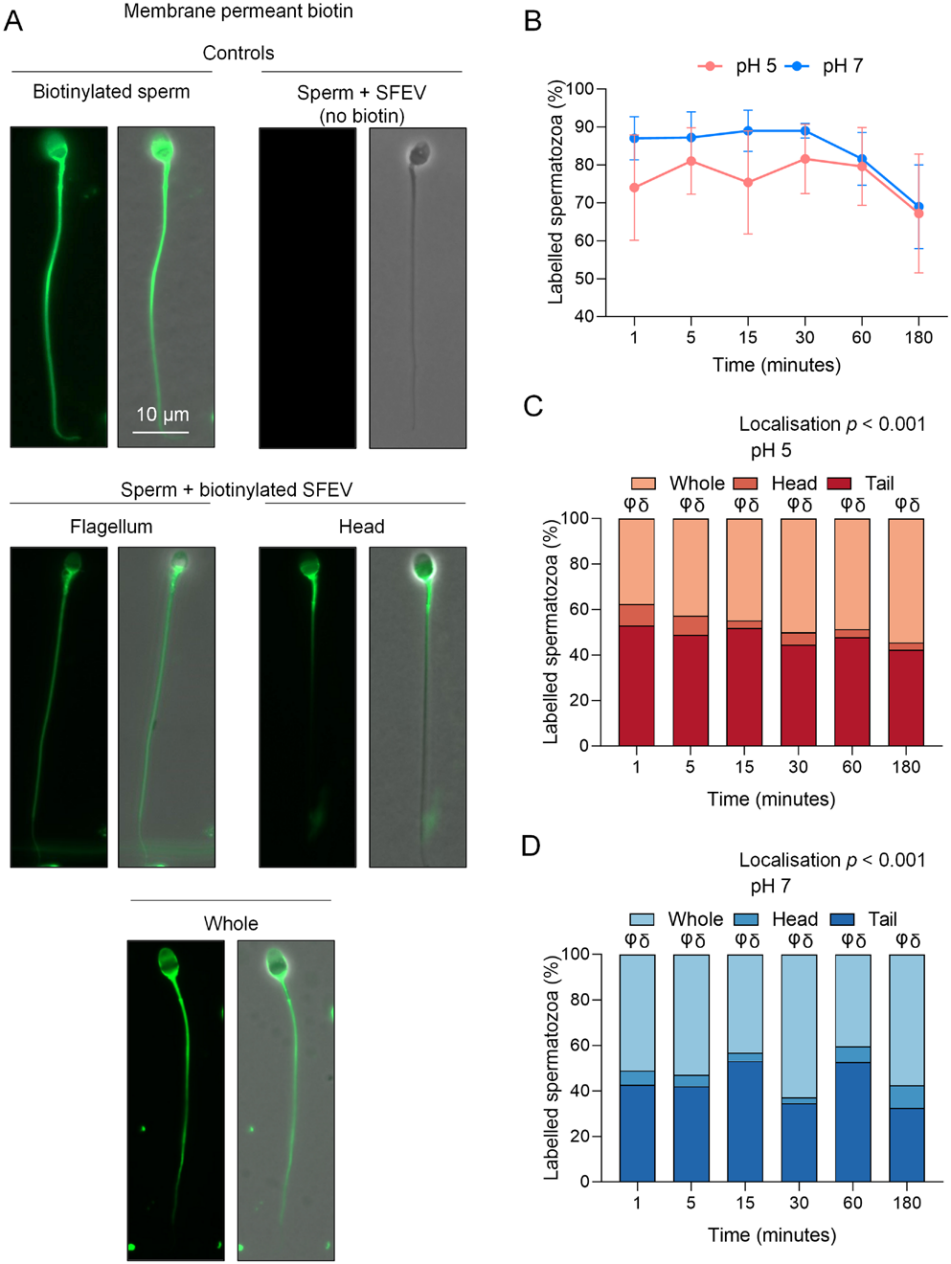
Assessment of the uptake and redistribution of biotinylated seminal fluid extracellular vesicle proteins following co-culture of human spermatozoa. Distinct from the previous experiment, proteins encapsulated within human seminal fluid extracellular vesicles (SFEVs) were labelled with a membrane-permeable derivative of biotin prior to co-incubation with spermatozoa over a time course of 3 h (with regular sampling at 1, 5, 15, 30, 60, and 180 min). Following incubation, spermatozoa were affinity-labelled with AlexaFluor 488 conjugated streptavidin to detect the uptake and fate of SFEV protein cargo. (A) Representative images illustrating the localisation patterns obtained following direct biotinylation of spermatozoa (i.e. no SFEVs), sperm incubation with unlabelled SFEVs (i.e. no biotin), and sperm incubation with SFEVs pre-labelled with membrane-permeable biotin reagent (scale bar = 10 µm). (B) The percentage of spermatozoa displaying the uptake of biotinylated proteins and the domains into which these proteins were distributed at (C) pH 5 and (D) pH 7 was determined by examining the fluorescence staining of a minimum of 100 cells per sample. All experiments were repeated using *n* = 5 independent biological replicates, with representative images being shown and graphical data in panels presented as (B) mean ±  s.e.m., and (C and D) stacked bar chart showing mean of each group. Data were assessed by 2-way ANOVA with Tukey’s multiple comparisons test (φ, *P* < 0.05 comparing sperm tail vs head labelling, δ, *P* < 0.05 comparing whole sperm vs head labelling). Differences in the overall effects of Localisation or Time are presented as text depicting the *P* value in each panel where a significant difference (*P <* 0.05) was detected.

As a prelude to investigating the influence of SFEV co-incubation on human sperm function, we first sought to establish the impact of pH on sperm viability under conditions that represent those encountered in the lower (i.e. pH 5) and upper (i.e. pH 7) portions of the female reproductive tract. As might be expected, sperm experienced a pH-dependent loss in viability when incubated at the acidic pH of 5 (Supplementary Figure 1). Sperm viability was immediately compromised at the initial assessment point in pH 5 media (i.e. *t* = 1 min) relative to that of cells suspended at the physiological pH of 7. Thereafter, spermatozoa experienced a progressive decline in viability such that by 300 min of incubation only 29% of the original population remained viable, compared to 87% in the pH 7 control group (*P* < 0.001; Supplementary Figure 1). No such loss of viability was observed in spermatozoa maintained in pH 7 BWW media (Supplementary Figure 1). In subsequent experiments, we utilised the pH 5 treatment to investigate whether SFEVs are capable of affording protection to buffer sperm against the negative impacts of the acidic environment they would encounter upon intromission into the female reproductive tract, as has been inferred from the results of independent research (Arienti *et al.* 1999). The results from these experiments were interpreted in the context of the effects of the two different pH environments (see Fig. 2 and 3 and accompanying text below).

We next examined the capacity of SFEVs to interact with a homologous population of spermatozoa by monitoring the kinetics of biotinylated protein cargo transfer between donor SFEVs and recipient spermatozoa. These studies featured a modified co-culture system previously developed for investigating the mechanistic basis of mouse sperm–epididymosome interactions (Zhou *et al.* 2019). Specifically, membrane impermeant (Fig. 2) and permeant (Fig. 3) variants of biotinylation reagents were utilised to label the SFEV protein cargo prior to co-culture with populations of spermatozoa isolated from the ejaculates of separate normozoospermic donors. Sperm-SFEVs were incubated at a ratio approximating 10 million sperm: 800 µg SFEV protein; a ratio selected from our preliminary studies that calculated the average EV protein content per ejaculate (average 3.5 mg). Although data from these pilot studies showed a wide range of EV concentrations, this ratio was selected to reflect the concentration of SFEVs that sperm from normozoospermic men (>39 million sperm/ejaculate) may encounter in a physiological setting. To control for the possibility of non-specific labelling due to non-reacted biotin reagent, these assays featured sperm samples that were directly labelled with biotin in the absence of SFEVs; a strategy that produced intense flagellum, mid-piece, and equatorial domain labelling accompanied by staining, albeit weaker, throughout the sperm head in virtually all spermatozoa for both membrane impermeant (Fig. 2A) and permeant variants of biotin (Fig. 3A). The effectiveness of quenching unreacted biotin was confirmed by conducting an incubation of quenched biotin with a naive population of sperm, revealing minimal labelling (data not shown). The possibility of artefactual labelling due to endogenous biotin signals was mitigated by direct staining of spermatozoa with streptavidin conjugated to Alexa Fluor 488 following incubation with non-biotinylated SFEVs, a strategy that confirmed no sperm staining (Figs. 2A and 3A).

Having established the specificity of SFEV-mediated biotin transfer, we exploited this system to track the transfer of SFEV proteins labelled with a membrane impermeant biotin reagent to permit assessment of the spatial and temporal characteristics of sperm-SFEV docking and adhesion (Fig. 2A). Affinity labelling of sperm cells with streptavidin conjugated to Alexa Fluor 488 was then used to determine the proportion of labelled sperm, as well as to visualise the localisation of labelling. These studies revealed substantial SFEV protein transfer occurs as early as 1 min post-incubation, with sperm populations held at both pH 5 (17.6%) and pH 7 (8.6%) exhibiting biotin labelling at this initial timepoint (Fig. 2B). Thereafter, over a co-incubation time course of 60 min, no significant increase in the percentage of biotin-labelled spermatozoa was seen irrespective of whether the cells were incubated with SFEVs at either pH 5 (maximum labelling of 22.4% at 5 min) or pH 7 (maximum labelling of 20.6% at 30 min) (Fig. 2B). Visualisation of the subcellular domain(s) targeted by SFEV cargo (Fig. 2A, C, D) at both pH 5 and pH 7 confirmed the selectivity of sperm–SFEV interactions, whereby the flagellum (encompassing both the mid- and principal-piece) was observed as the preferential site for SFEV biotinylated protein deposition across the 60 min co-incubation period (Fig. 2C and D). However, a subset of cells in each population of spermatozoa also showed biotin labelling distributed over the entire cell or restricted primarily to the head (in some cells, a combination of head and midpiece staining was observed) (Fig. 2C and D), Quantification of these labelling patterns demonstrated that at each time point in pH 5 media, flagellum labelling was significantly increased compared to both whole sperm and head labelling (*P* < 0.05). In contrast, at pH 7, while flagellum labelling was the preferential site of SFEV biotinylated protein deposition at each time point, the prevalence of this labelling pattern was significantly increased only compared to whole sperm labelling (*P* < 0.05), and not head labelling (Fig. 2 A and D). Notably, the spatial distribution of sperm biotin labelling was not significantly altered during the 60 min of sperm-SFEV co-incubation (Fig. 2C and D).

To complement the investigation of the transfer of surface-exposed SFEV proteins to spermatozoa (i.e. those labelled with membrane-impermeable biotin), we also sought to track trafficking of encapsulated SFEV proteins by labelling with a membrane-permeant biotin derivative (Fig. 3); a strategy we have previously used to characterise the uptake and redistribution of EV proteins beyond the initial site of EV adhesion (Zhou *et al.* 2019). The initial kinetics of encapsulated SFEV protein transfer appeared similar to those of membrane SFEV proteins, with protein deposition observed in spermatozoa as soon as 1 min post-incubation. However, the relative efficacy of protein transfer was dramatically higher, such that an average of 74% (pH 5) and 87% (pH 7) of the sperm cells exhibited biotin labelling at this initial timepoint (Fig. 3B). Thereafter, we failed to observe any additional increase in sperm labelling over the 180-min time course of the study, irrespective of the pH of the incubation medium (Fig. 3B). Similarly, the spatial distribution of SFEV-encapsulated proteins appeared to follow a similar pattern to that of SFEV membrane proteins at both pH conditions, with the notable exception that both flagellum and whole sperm biotin labelling were the predominant domains visualised, and were significantly increased at each time point compared to the small population of spermatozoa that exhibited head or a combination of head and midpiece labelling (*P <* 0.05, Fig. 3C and D).

### Seminal fluid extracellular vesicles exert minimal influence on human sperm motility, capacitation or acrosomal exocytosis

Notwithstanding some evidence to the contrary, the balance of literature has implicated SFEVs in the support of sperm development, function, and/or protection from the acidic microenvironment encountered within the vagina (Arienti *et al.* 1997, Palmerini *et al.* 1999, Palmerini *et al.* 2003, Arienti *et al.* 2004, Park *et al.* 2011, Murdica *et al.* 2019*b*, Wang *et al.* 2022). Accordingly, in view of the productive interactions recorded between human sperm and SFEVs (Figs. 2 and 3), we next sought to explore the functional implications of these interactions via assessment of key parameters of sperm function. Specifically, we assessed the influence of SFEVs on sperm motility parameters (using CASA) and the ability to undergo capacitation and complete an ionophore-induced acrosome reaction over a 300-min time course, in which high-quality spermatozoa collected following seminal fluid liquefaction were co-incubated with SFEVs isolated from distinct donors at a ratio of 10 million sperm cells per 800 µg of SFEV protein.

**Figure 4:**
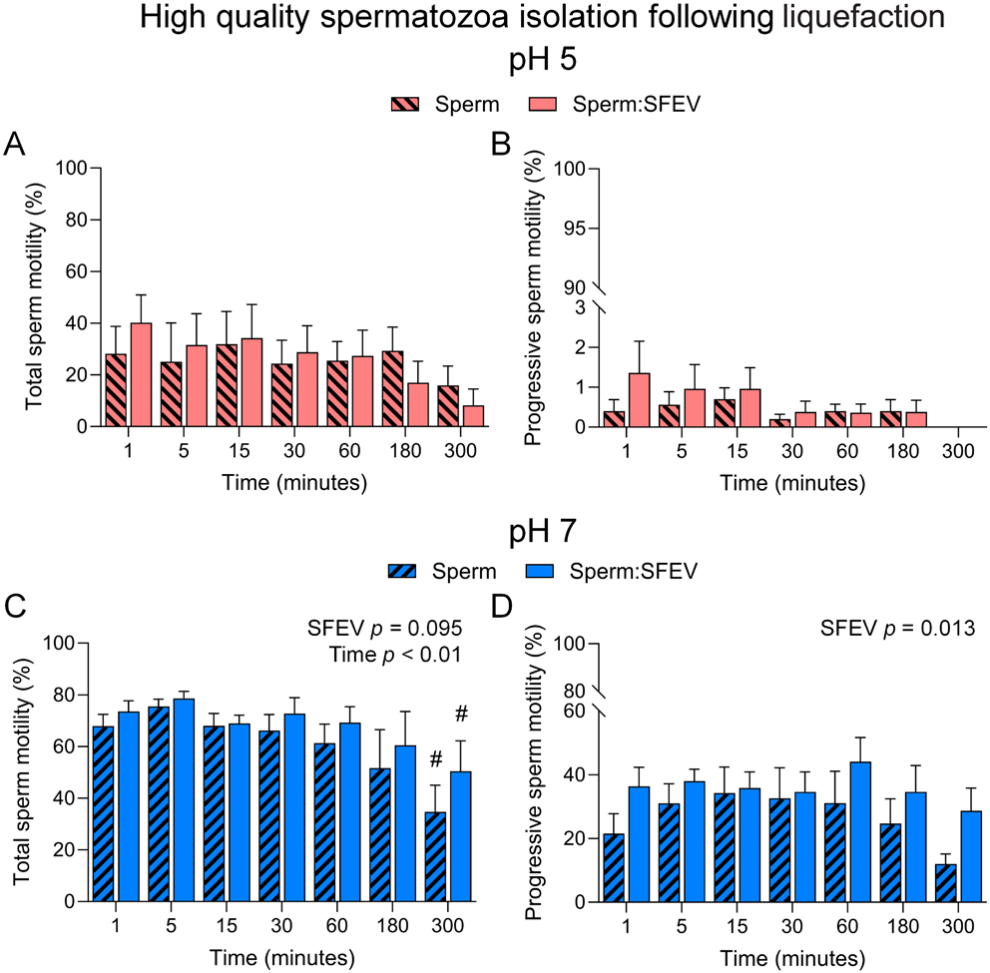
Seminal fluid extracellular vesicles (SFEVs) have minimal influence on the function of spermatozoa. Human spermatozoa were fractionated over a discontinuous Percoll density gradient and high-quality sperm were resuspended in non-capacitating Biggers, Whitten, and Whittingham medium (BWW) buffered to either (A and B) pH 5 or (C and D) pH 7 and incubated for 1, 5, 15, 30, 60, 180, or 300 min. At regular intervals throughout the extended co-incubation, a portion of the sperm suspension was sampled and assessed for (A and C) total motility and (B and D) progressive motility using objective computer-assisted sperm analysis (CASA) counting a minimum of 200 cells per sample. All experiments were repeated using *n* = 5 independent biological replicates. Graphical data are presented as mean ±  s.e.m. Data were assessed by 2-way ANOVA with Tukey’s multiple comparisons test. Differences in the overall effects of SFEV or time are presented as text depicting the *P* value in each panel where a significant difference (*P <* 0.05) was detected. Significant changes at individual time points are depicted using symbols (#, *P* < 0.05 difference within treatment group compared to earlier time points).

Consistent with compromised sperm viability at pH 5 (Supplementary Figure 1), spermatozoa immediately exhibited lower overall and progressive motility when incubated in BWW media buffered to pH 5 (Fig. 4A and B) vs pH 7 (Fig. 4C and D). No further significant compromise in pH 5 buffered media was documented in either total (Fig. 4A), or progressive (Fig. 4B) motility over the remainder of the 300-min incubation period. Moreover, supplementation of the incubation medium with SFEVs in pH 5 media failed to elicit any protective effect against this acidic environment in contrast to reports by others (Arienti *et al.* 1999). Further assessment of spermatozoa kinematic parameters following SFEV incubation using CASA showed no overall impact of SFEV across the time-course or at individual time points (Supplemental Figure 2). Due to the low progressive motility in spermatozoa incubated in pH 5 buffered media (average 0.5% progressive motility, Fig. 4B), spermatozoa kinematic parameters were not further explored for this population of cells.

When considering motility data collected for spermatozoa incubated in SFEVs at pH 7, we noted a reduction in total sperm motility across the time-course. However, this effect was conserved in populations of spermatozoa incubated both in the presence and absence of SFEVs (Fig. 4C and D). This was particularly notable at 300 min, where motility was significantly lower in sperm incubated in the presence and absence of SFEVs compared to the 1, 5, 15, and 30 min time points (*P* < 0.05, Fig. 4C). These changes were not apparent in the progressively motile sperm population (Fig. 4D). Although SFEVs failed to elicit an increase in motile (Fig. 4C) or progressively motile (Fig. 4D) sperm cells at individual time points, 2-way ANOVA analyses showed that across the time-course, there was an overall increase in total (*P* = 0.095, Fig. 4C) and progressive (*P* < 0.05, Fig. 4D) motility attributable to SFEV co-incubation.

**Figure 5:**
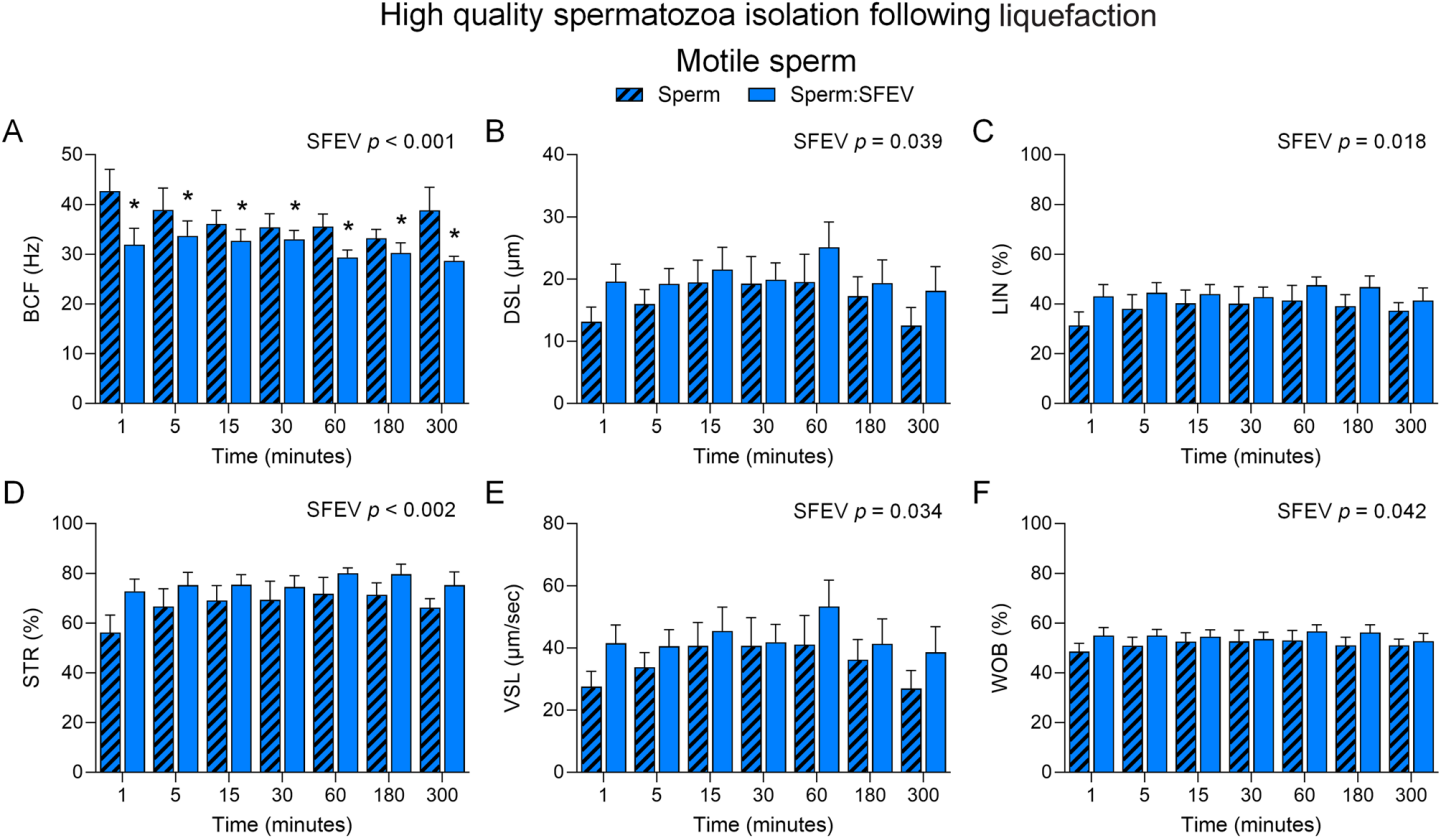
Seminal fluid extracellular vesicles (SFEVs) have minimal influence on spermatozoa motility parameters in a neutral pH (pH 7) environment. Human spermatozoa were fractionated over a discontinuous Percoll density gradient and high-quality sperm were resuspended in non-capacitating Biggers, Whitten, and Whittingham medium (BWW) buffered to pH 7 and incubated for 1, 5, 15, 30, 60, 180, or 300 min with SFEVs as described in Fig. 4. At regular intervals throughout the extended co-incubation, a portion of the sperm suspension was sampled and assessed for motility parameters using objective computer-assisted sperm analysis (CASA) counting a minimum of 200 cells per sample. Parameters assessed included (A) Beat cross frequency (BCF, Hz), (B) Distance straight line (DSL, µm), (C) Linearity (LIN, %), (D) Straightness (STR, %), (E) Straight-line velocity (VSL, µm/second), and (F) Wobble (WOB, %). All experiments were repeated using *n* = 5 independent biological replicates. Graphical data are presented as mean ±  s.e.m. Data were assessed by 2-way ANOVA with Tukey’s multiple comparisons test. Differences in the overall effects of SFEV or Time are presented as text depicting the *P* value in each panel where a significant difference (*P <* 0.05) was detected. Significant changes at individual time points are depicted using symbols (*P < 0.05 vs same time point comparing sperm: SFEV and sperm alone groups).

**Figure 6:**
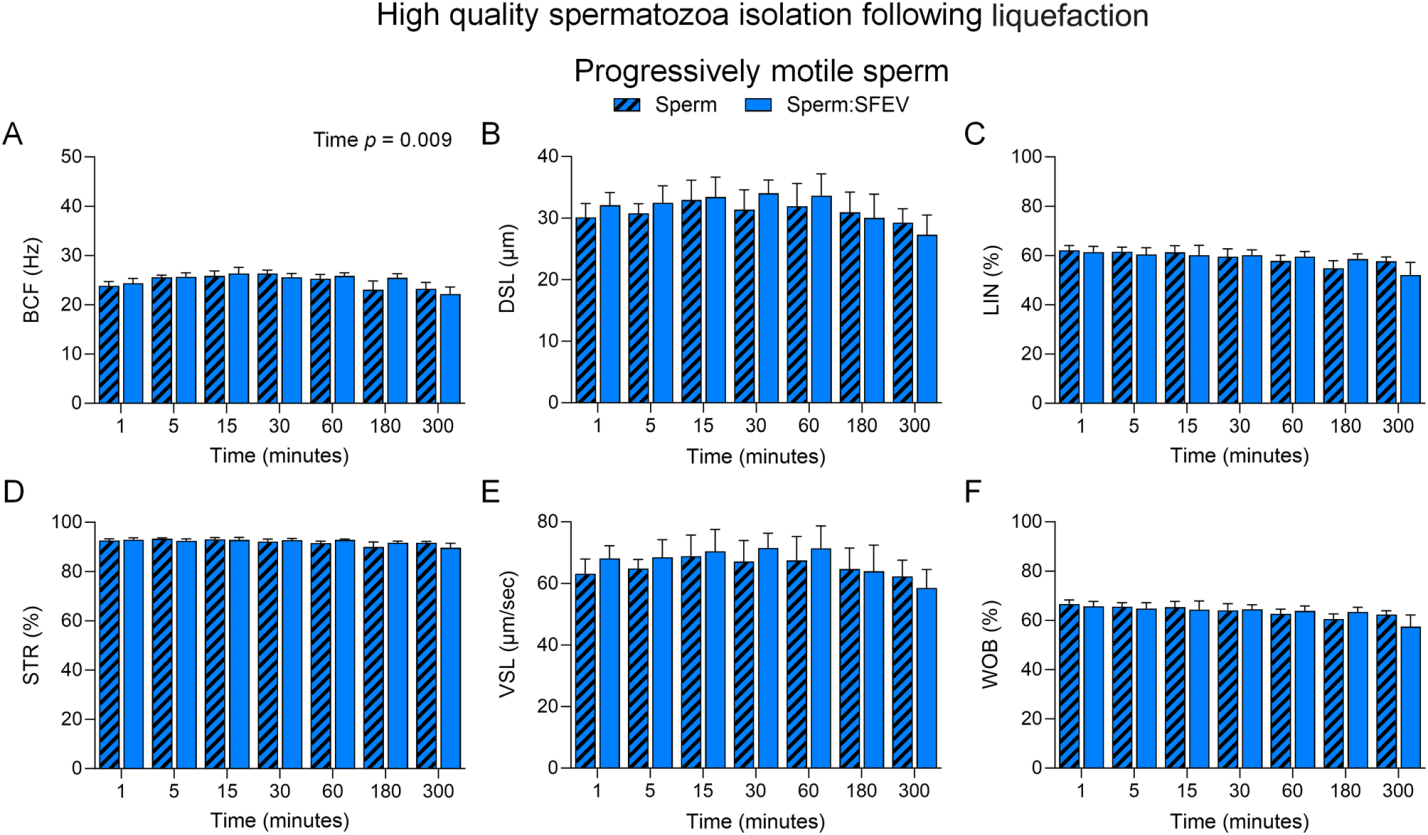
Seminal fluid extracellular vesicles (SFEVs) do not influence spermatozoa progressive motility parameters in a neutral pH (pH 7) environment. Human spermatozoa were fractionated over a discontinuous Percoll density gradient and high-quality sperm were resuspended in non-capacitating Biggers, Whitten, and Whittingham medium (BWW) buffered to pH 7 and incubated for 1, 5, 15, 30, 60, 180, or 300 min with SFEVs as described in Fig. 4. At regular intervals throughout the extended co-incubation, a portion of the sperm suspension was sampled and assessed for progressive motility kinetic parameters using objective computer-assisted sperm analysis (CASA) counting a minimum of 200 cells per sample. Parameters assessed included (A) Beat cross frequency (BCF, Hz), (B) Distance straight line (DSL, µm), (C) Linearity (LIN, %), (D) Straightness (STR, %), (E) Straight-line velocity (VSL, µm/second), and (F) Wobble (WOB, %). All experiments were repeated using *n* = 5 independent biological replicates. Graphical data are presented as mean ±  s.e.m. Data were assessed by 2-way ANOVA with Tukey’s multiple comparisons test. Differences in the overall effects of SFEVs or time are presented as text depicting the *P* value in each panel where a significant difference (*P <* 0.05) was detected.

Extending this analysis to include consideration of the detailed sperm motility kinematics generated by CASA (Figs. 5 and 6, Supplemental Figure 3), we noted a significant decrease in Beat cross frequency (BCF) in populations of motile spermatozoa incubated with SFEVs at each timepoint (*P* < 0.05, Fig. 5A). While no other parameters showed significant changes at individual time points, there was an overall increase in parameters including distance straight line (DSL, Fig. 5B, *P* < 0.05), linearity (LIN, Fig. 5C, *P* < 0.05), straightness (STR, Fig. 5D, *P* < 0.05), straight line velocity (VSL, Fig. 5E*P* < 0.05) and wobble (WOB, Fig, 5F, *P* < 0.05) attributable to SFEVs in the population of total motile spermatozoa as measured by 2-way ANOVA. No changes were recorded for other CASA kinematic parameters assessed in the population of total motile spermatozoa (Supplemental Figure 3A-E). In contrast, CASA kinematic assessment of progressively motile sperm populations showed no impact of SFEVs on any measured sperm kinematic parameter (Fig. 6, Supplemental Figure 3F-J).

**Figure 7:**
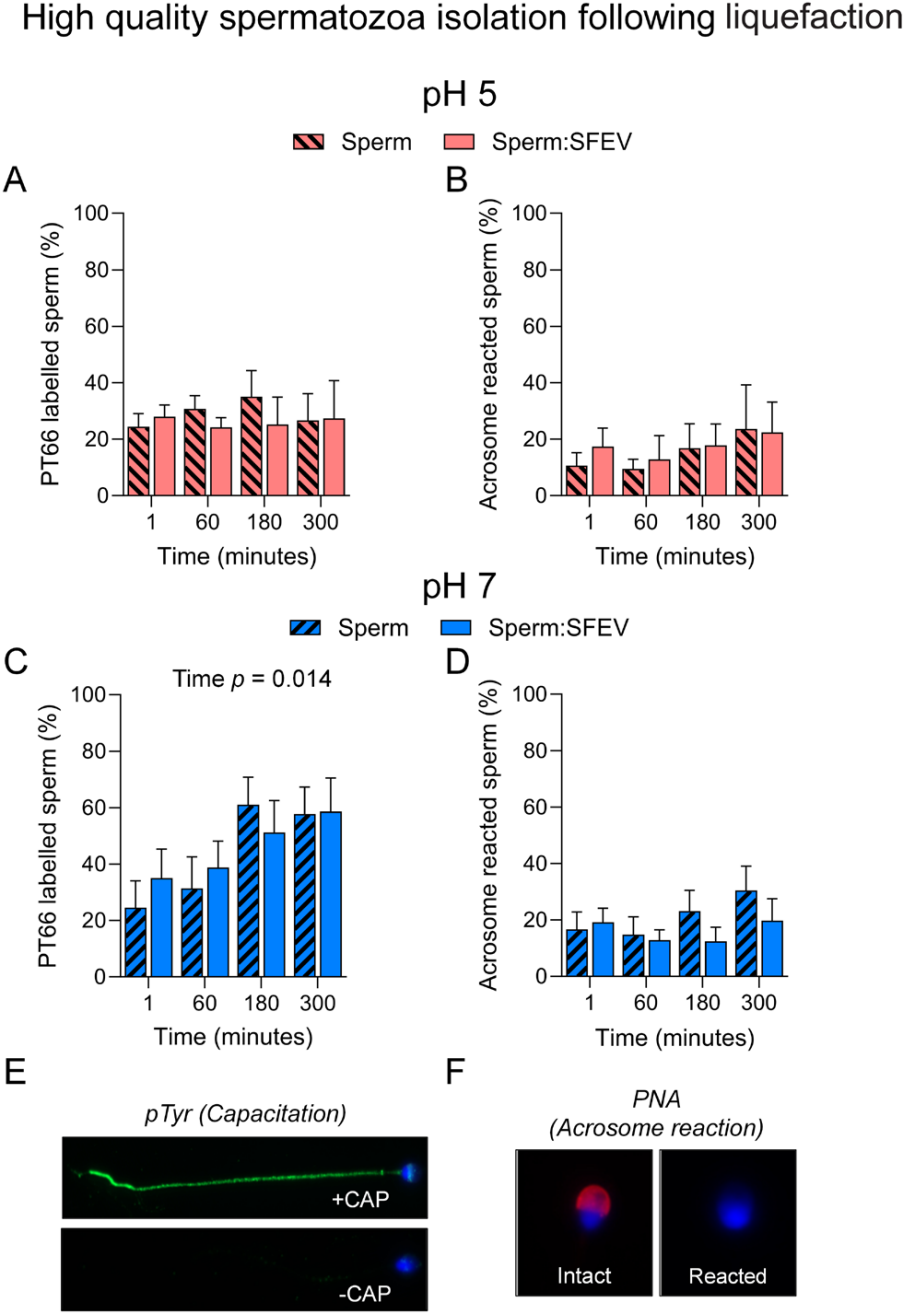
Seminal fluid extracellular vesicles (SFEVs) do not influence capacitation or acrosome reaction rates of spermatozoa isolated from normozoospermic donors. Human spermatozoa were fractionated over a discontinuous Percoll density gradient and high-quality sperm were resuspended in non-capacitating Biggers, Whitten, and Whittingham medium (BWW) buffered to either (A and B) pH 5 or (C and D) pH 7 and incubated for 1, 60, 180, or 300 min. At regular intervals throughout the extended co-incubation, a portion of the sperm suspension was sampled and capacitation status and the ability to complete an acrosome reaction were assessed using immunofluorescence-based assays. (A and C) Sperm were immunostained with anti-phosphotyrosine antibodies to determine capacitation status, or (B and D) incubated with the calcium ionophore A23187 to assess their capacity to undergo an ionophore-induced acrosome reaction; with representative images depicted from positive and negative controls for (E) capacitation (pentoxifylline-driven) and (F) acrosome reaction (A23187-driven following capacitation). Graphical data are presented as mean ±  s.e.m. Data were assessed by 2-way ANOVA with Tukey’s multiple comparisons test. Differences in the overall effects of SFEVs or time are presented as text depicting the *P* value in each panel where a significant difference (*P <* 0.05) was detected.

Having only shown subtle effects of SFEVs in the modulation of sperm motility, we next assessed the ability of sperm to complete capacitation (Fig. 7A, C, E) and undergo an ionophore-induced acrosome reaction (Fig. 7B, D, F); two functional parameters previously reported as displaying sensitivity to SFEVs (Palmerini *et al.* 1999, Palmerini *et al.* 2003, Park *et al.* 2011, Murdica *et al.* 2019*b*, Wang *et al.* 2022). When suspended in BWW buffered to pH 5, the duration of incubation did not affect either the induction of sperm capacitation (Fig. 7A) or acrosomal exocytosis (Fig. 7B) irrespective of the presence or absence of SFEVs in the culture media. By comparison, BWW buffered to a more physiological pH of 7 readily supported a time-dependent increase in capacitation, as assessed via phosphotyrosine labelling, although this response was similarly not influenced by SFEV exposure (Fig. 7C). Additionally, SFEVs did not elicit any significant change in the number of sperm undergoing an acrosome reaction over the course of co-incubation (Fig. 7D).

**Figure 8:**
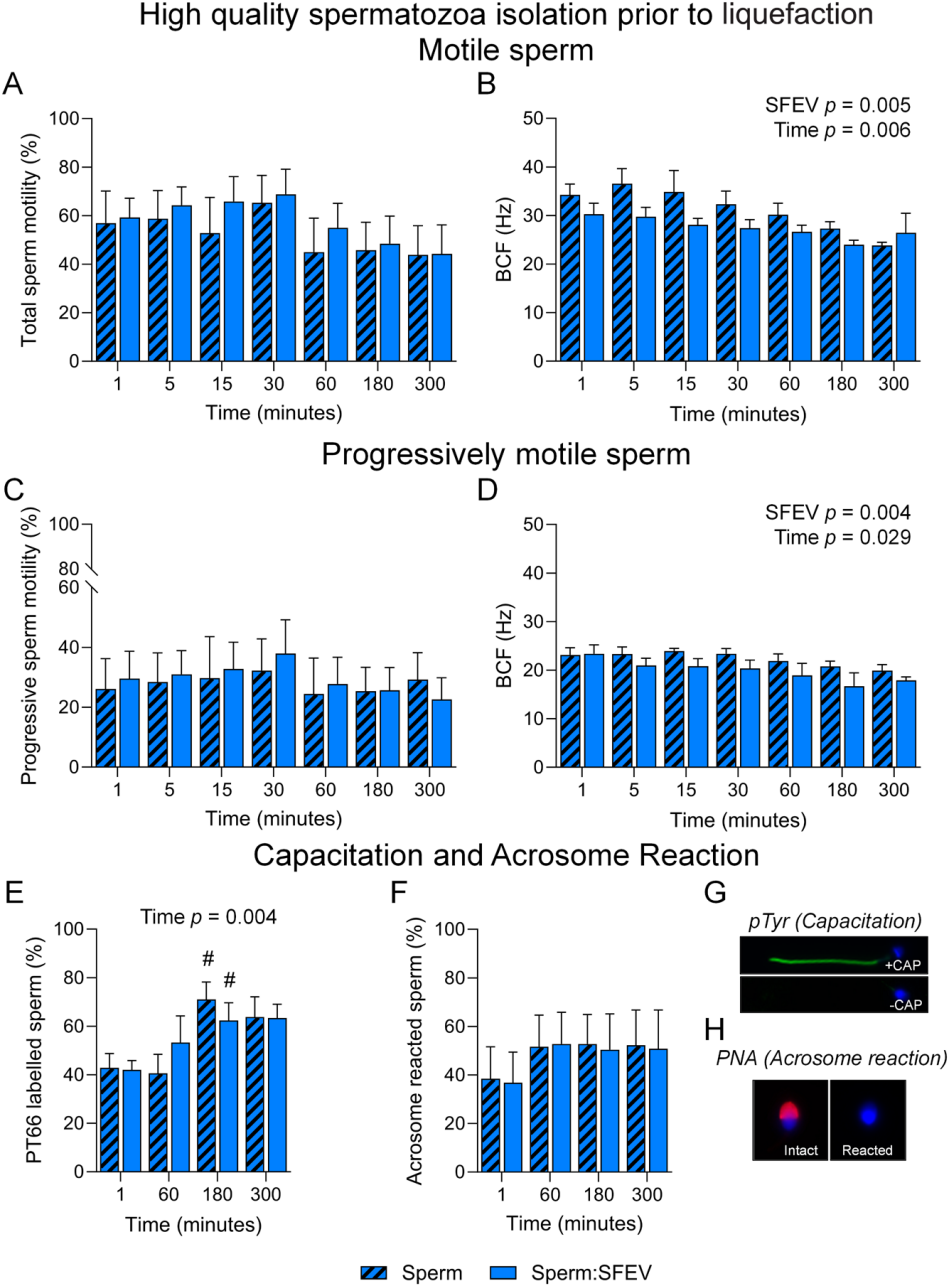
Seminal fluid extracellular vesicles (SFEVs) have minimal influence on the functional parameters of high-quality sperm collected prior to liquefaction. Human spermatozoa collected prior to liquefaction were fractionated over a discontinuous Percoll density gradient and high-quality sperm were resuspended in non-capacitating Biggers, Whitten, and Whittingham medium (BWW) buffered to pH 7 and incubated for a period of 300 min. At regular intervals throughout the extended co-incubation, a portion of the sperm suspension was sampled and assessed for motility parameters (A–D), capacitation status (E and G), and ability to complete an acrosome reaction (F and H). Assessment of sperm parameters utilised objective computer-assisted sperm analysis (CASA), counting a minimum of 200 cells per sample. Data presented are (A) Total sperm motility (%), (B) Beat cross frequency (BCF, Hz) of the motile sperm population, (C) Progressive sperm motility (%), and (D) BCF (Hz) of the progressively motile sperm population. Assessment of capacitation status and the ability to complete an acrosome reaction used immunofluorescence-based assays. (E and G) Sperm were immunostained with anti-phosphotyrosine antibodies to determine capacitation status, or (F and H) incubated with the calcium ionophore A23187 to assess their capacity to undergo an ionophore-induced acrosome reaction; with representative images depicted from positive and negative controls for (G) capacitation (pentoxifylline-driven) and (H) acrosome reaction (A23187-driven following capacitation). Graphical data are presented as mean ±  s.e.m. Data were assessed by 2-way ANOVA with Tukey’s multiple comparisons test. Differences in the overall effects of SFEV or Time are presented as text depicting the *P* value in each panel where a significant difference (*P <* 0.05) was detected. Significant changes at individual time points are depicted using symbols (#, *P* < 0.05 difference within treatment group compared to the 1-mintime point).

The limited influence of SFEVs on the functional capacity of human spermatozoa raised several possibilities. One possibility was that recipient high-quality sperm populations had become saturated with SFEVs during the process of liquefaction. To address this, we isolated spermatozoa from semen samples prior to semen liquefaction (within 15 min of ejaculation) and assessed the functional consequences of sperm – SFEV interactions in high-quality spermatozoa. In these studies, in both motile (Fig. 8A and B) and progressively motile (Fig. 8C and D) sperm populations, we observed no change in motility over the 300-min time course (Fig. 8A and C), but did observe an overall decrease in BCF attributable to SFEVs as measured by 2-way ANOVA (Fig. 8B and D). No further changes were observed in either sperm population following detailed CASA kinematic assessment, with the exception of an overall decrease in linearity and straightness as measured by 2-way ANOVA that was attributed to SFEVs in progressively motile sperm populations (Supplemental Figure 4). Additionally, while there was a time-dependent increase in sperm capacitation over the incubation period (Fig. 8E), this response was not influenced by SFEV exposure (Fig. 8E and G), nor was acrosome reaction status altered (Fig. 8F and H).

**Figure 9:**
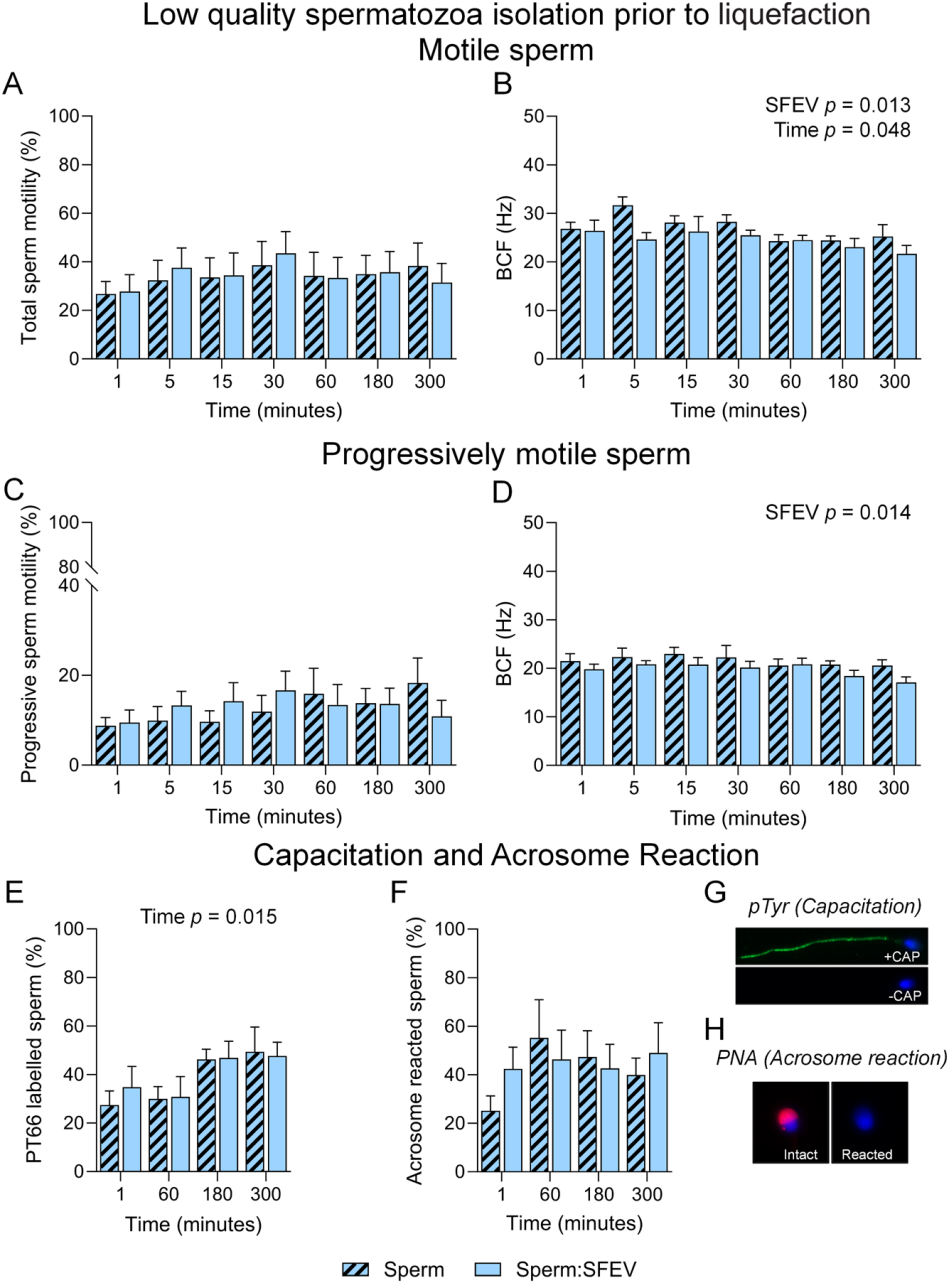
Seminal fluid extracellular vesicles (SFEVs) have minimal influence on the functional parameters of low-quality sperm collected prior to liquefaction. Human spermatozoa collected prior to liquefaction were fractionated over a discontinuous Percoll density gradient and low-quality sperm were resuspended in non-capacitating Biggers, Whitten, and Whittingham medium (BWW) buffered to pH 7 and incubated for a period of 300 min. At regular intervals throughout the extended co-incubation, a portion of the sperm suspension was sampled and assessed for motility parameters (A–D), capacitation status (E and G), and ability to complete an acrosome reaction (F and H). Assessment of sperm parameters utilised objective computer-assisted sperm analysis (CASA), counting a minimum of 200 cells per sample. Data presented are (A) Total sperm motility (%), (B) Beat cross frequency (BCF, Hz) of the motile sperm population, (C) Progressive sperm motility (%), and (D) BCF (Hz) of the progressively motile sperm population. Assessment of capacitation status and the ability to complete an acrosome reaction used immunofluorescence-based assays. (E and G) Sperm were immunostained with anti-phosphotyrosine antibodies to determine capacitation status, or (F and H) incubated with the calcium ionophore A23187 to assess their capacity to undergo an ionophore-induced acrosome reaction; with representative images depicted from positive and negative controls for (G) capacitation (pentoxifylline-driven) and (H) acrosome reaction (A23187-driven following capacitation). Graphical data are presented as mean ±  s.e.m. Data were assessed by 2-way ANOVA with Tukey’s multiple comparisons test. Differences in the overall effects of SFEV or Time are presented as text depicting the *P* value in each panel where a significant difference (*P <* 0.05) was detected.

A second possibility that we considered was that SFEVs may have a more profound effect on low-quality spermatozoa. To assess this, we collected low-quality spermatozoa partitioning at the interface of the 40% and 80% Percoll gradient and assessed their functional status following SFEV incubation. Consistent with high-quality spermatozoa (Fig. 8), both overall motility (Fig. 9A and B) and progressive motility of low-quality spermatozoa (Fig. 9C and D) were not altered over the 300-min time course (Fig. 9A and C). We did, however, note an overall decrease in BCF as measured by 2-way ANOVA as being attributable to SFEVs (Fig. 9B and D). No further changes were observed in either sperm population following detailed CASA kinematic assessment (Supplemental Figure 4). Finally, while there was a time-dependent increase in capacitation over the 300-min incubation (Fig. 8E), SFEVs did not elicit any changes in capacitation (Fig. 8E and G), or acrosome reaction (Fig. 8F and H) status among the low-quality sperm population.

## Discussion

Seminal fluid is widely recognised as harbouring one of the most abundant EV populations of any body fluid (Vojtech *et al.* 2014, Mercadal *et al.* 2020, Tamessar *et al.* 2021). Owing to contributions from the multiple tissues that comprise the male reproductive system, SFEVs display considerable heterogeneity in terms of both physical properties and potential function (Aalberts *et al.* 2012, Belleannee *et al.* 2013, [Bibr bib13][Bibr bib25]). With regard to the latter, SFEVs have attracted interest as putative regulators of reproductive events as diverse as the modulation of sperm function through to conditioning the peri-conceptional environment within the female reproductive tract (Kelly *et al.* 1991, Arienti *et al.* 1999, Carlsson *et al.* 2003, Park *et al.* 2011, Murdica *et al.* 2019*a*,*b*, Rodriguez-Caro *et al.* 2019, Vojtech *et al.* 2019, George *et al.* 2020). However, while their affinity for spermatozoa is well established, comparatively less is known of the mechanistic basis by which SFEVs recognise and divest their cargo to the recipient sperm population. There also remains some contention regarding the spectrum of functional consequences and degree of effect arising from these interactions. This study was formulated to address these questions via the use of a tractable *in vitro* co-culture system to assess the kinetics, spatial distribution, and physiological influence of human spermatozoa–SFEV interactions. The major findings of this work were that SFEVs rapidly interact and deliver their protein cargo to human spermatozoa. Notwithstanding the dispersal of this SFEV cargo throughout the sperm cell, with the primary site of SFEV protein cargo deposition being the flagellum, EVs appeared capable of eliciting only subtle modifications to the function of sperm.

Whilst substantial progress has been made towards understanding EV biology over the last decade, the selection of consistent approaches for EV isolation has been an ongoing challenge (Van Deun *et al.* 2017, Thery *et al.* 2018, Mercadal *et al.* 2020). This is despite the choice of isolation method being one of the most important considerations in EV research since the ability to accurately ascribe functional roles to these entities is dictated by the degree of EV enrichment, the heterogeneity of the EV population, and their relative concentration; all of which have the potential to impact the interpretation of experiments and the assignment of biological activity (Thery *et al.* 2018). In recognition of these limitations, we adapted a density gradient isolation method previously developed (Zhou *et al.* 2019, Trigg *et al.* 2021) in accordance with MISEV guidelines (Thery *et al.* 2018), to enrich EVs from human seminal fluids. The recovered SFEVs displayed the expected physical properties (i.e. buoyant density, diameter, bilayer) and established EV markers, confirming the utility of the purification strategy and the suitability of the SFEVs to address our experimental aims.

The notion that spermatozoa interact with SFEVs implies that this association and the subsequent exchange of cargo must occur very rapidly, either coinciding with sperm passage through the ejaculatory ducts or immediately after insemination into the lower portion of the female reproductive tract, as sperm begin to traverse the female reproductive tract to the site of oocyte fertilisation in the ampulla of the fallopian tube (Arienti *et al.* 1997, Carlini *et al.* 1997, Arienti *et al.* 1999, Arienti *et al.* 2004). Accordingly, we documented the transfer of biotin-labelled protein cargo from SFEVs to spermatozoa within as little as 1 min of co-incubation; thereafter, we failed to record any further exchange, such that the number of biotinylated sperm remained static over a prolonged co-incubation period of 60–180 min. These kinetics resemble those recorded for the interaction between mouse spermatozoa and a purified population of epididymosomes (i.e. EVs originating in the epididymis) (Zhou *et al.* 2019). However, both the distribution of biotinylated proteins and proportion of labelled cells differed between these studies, potentially reflecting a different distribution of EV receptors on mouse versus human spermatozoa. Unlike epididymosomes, which predominantly bound to the post-acrosomal region of the mouse sperm head (Zhou *et al.* 2019), human SFEVs appeared to concentrate within the sperm flagellum but were also found distributed over the entire cell in at least a subset of spermatozoa. These data may be attributed to the heterogeneous population of EVs present in seminal fluid, with potential contributions from the testes, epididymis, and male accessory glands (i.e. prostate, seminal vesicles, and bulbourethral glands) that may interact and deliver their encapsulated cargo to unique sperm domains (Fabiani *et al.* 1994, Belleannee *et al.* 2013, Hoog & Lotvall 2015, Tamessar *et al.* 2021).

A curious finding in the current study was that the maximum number of spermatozoa that received SFEV cargo labelled with a membrane-impermeable biotin reagent peaked at ~20% of the population, in line with other studies using alternative membrane/lipid labelling reagents (Arienti *et al.* 1997, Carlini *et al.* 1997, Arienti *et al.* 2004, Murdica *et al.* 2019*b*). In contrast, considerably more spermatozoa (~80%) were seen to take up SFEV cargo labelled with a membrane-permeable biotin derivative. This proportion aligns with independent immunogold electron microscopy analysis, where SFEV-derived CD38 molecule and ryanodine receptors (RyR) were detected in 80% of sperm following fusion with SFEVs (Park *et al.* 2011). The purpose of tracking the transfer of differentially biotinylated SFEV proteins is that it allows the discrimination of SFEV tethering/adhesion (i.e. via labelling of those proteins exposed on the SFEV surface with membrane-impermeable biotin) as opposed to downstream uptake and redistribution of SFEV cargo (i.e. via labelling of those proteins encapsulated within the SFEVs with membrane-permeable biotin). It might reasonably be expected that a greater or equivalent number of spermatozoa should take up membrane-impermeable biotin after co-incubation with SFEVs. Although the resolution of this apparent dichotomy awaits further investigation, one potential explanation rests with the nature of sperm–EV interactions. We have previously postulated a ‘kiss-and-run’ model (Watanabe & Boucrot 2017) whereby EVs transiently dock and fuse with the sperm membrane before fusion pore closure and release (Zhou *et al.* 2019). If this model is correct, then the membrane-impermeable biotin labelling may be registering tethered SFEVs that are in the process of actively transferring their cargo, while membrane-permeable biotin records the legacy of those cells that have accumulated SFEV cargo over the course of the co-incubation. Alternatively, there may be different subsets of sperm with low and high rates of uptake of SFEVs, such that those labelled by membrane impermeable biotin may have a diminished capacity to rapidly internalise SFEV cargo. Depending on the impact of that cargo, this may affect their destiny in the female reproductive tract – for example, their capacity to avoid sequestration by infiltrating neutrophils (Alghamdi *et al.* 2004).

Irrespective of the mechanism of sperm-SFEV interaction, the fact that this process facilitates protein transfer provides credence to previous studies that have implicated SFEVs in the modulation of sperm function (Arienti *et al.* 1997, Palmerini *et al.* 1999, Palmerini *et al.* 2003, Arienti *et al.* 2004, Park *et al.* 2011, Murdica *et al.* 2019*b*). Indeed, the most recent proteomic analyses of human SFEV subpopulations demonstrate that they encapsulate abundant proteomic cargo, with some 1500-4000 SFEV proteins having been identified (Murdica *et al.* 2019*a*, Lin *et al.* 2019, Zhang *et al.* 2020, Wang *et al.* 2022). Moreover, proteins identified as being carried by SFEVs, including CD38 (Park *et al.* 2011), cysteine rich secretory protein 1 (CRISP1) (Murdica *et al.* 2019*b*), glycodelin (Murdica *et al.* 2019*a*), and transient receptor potential cation channel subfamily V member 6 (TRPV6) (Lin *et al.* 2019) are speculated to influence sperm functional changes in the female reproductive tract. Given the large number of proteins detected in SFEVs, it is likely that other as-yet unidentified cargo able to influence sperm function are carried by these vesicles. Thus, studies to identify SFEV factors delivered to sperm are essential, particularly given the ‘kiss-and-run’ model brings forth the possibility that SFEVs selectively transfer cargo to sperm, or even selectively remove sperm cargo that is no longer required (Leahy *et al.* 2020, Roca *et al.* 2022).

While we remain uncertain whether sperm–SFEV interactions facilitate the transfer of all SFEV bioactive cargo to recipient spermatozoa, our study did reveal that the majority of proteins relayed to sperm via SFEVs appeared to compartmentalise within the sperm flagellum. This domain not only accommodates the sperm motility apparatus but also exclusively houses the sperm mitochondria and the bulk of their glycolytic machinery (du Plessis *et al.* 2015) - all of which are required to aid sperm migration through the female reproductive tract. Despite the potential implications of these combined data in terms of SFEV modulation of sperm metabolic activity, we found minimal evidence that sperm motility profiles were responsive to SFEV co-incubation. Notably, there was an overall increase in total and progressive sperm motility that was attributable to SFEV co-incubation with high-quality sperm at a neutral pH, but these were only subtle changes. Similarly, we noted subtle modifications to kinematic sperm motility parameters objectively measured via CASA; changes that closely mimicked parameters observed in the progressively motile population.

We considered the possibility that low-quality and high-quality spermatozoa have different responses to SFEVs. This is an important consideration as many studies showing increases in motility following sperm: SFEV co-incubation did not separate high- and low-quality spermatozoa (Fabiani *et al.* 1994, Wang *et al.* 2022). Further, normozoospermic sperm with a basal progressive motility of 40% were shown to be less responsive to SFEVs compared to sperm with a lower basal progressive motility of 10% (Murdica *et al.* 2019*b*). Consistent with our observations following co-incubation with high-quality spermatozoa, we only noted subtle modifications to sperm kinematic parameters in low-quality spermatozoa. Thus, the subtle changes attributable to SFEV observed in our dataset appear to apply to both low- and high-quality sperm populations.

In view of these data, our analysis was extended to examine other potential roles for SFEVs, with a particular focus on the protection they may afford sperm cells against the different microenvironments encountered within the female reproductive tract. This notion informed our investigations of sperm–SFEV interactions at pH 5 and pH 7, conditions that mimic the pH in the lower and upper portions of the female reproductive tract, respectively. Highlighting the potential of pH to influence the nature and/or efficacy of sperm–SFEV interactions, previous studies have reported distinct patterns of SFEV binding to human spermatozoa, whereby acidic incubation conditions promoted interaction with either the sperm neck (Arienti *et al.* 1997, Park *et al.* 2011) or the entire sperm surface (Arienti *et al.* 2004), while equivalent neutral buffered media has been consistently shown to support binding over the entire sperm surface (Arienti *et al.* 1997, Hoog & Lotvall 2015, Wang *et al.* 2022).

Using the transfer of biotinylated SFEV proteins to sperm as a readout of sperm–SFEV interactions, we failed to record any influence of pH on either the kinetics, efficacy, or sperm domains targeted for SFEV interaction. We also found no evidence that SFEV interactions were able to prolong sperm viability in either acidic or neutral pH media, nor accelerate or suppress the induction of sperm capacitation or acrosomal exocytosis; functional endpoints that provide an indication of the fertilisation competence of the sperm population. These results are incongruous with earlier work demonstrating that EVs can counteract the loss of sperm motility that accompanies their suspension in acidic conditions mimicking vaginal pH (Arienti *et al.* 1999), and studies showing that SFEVs can promote sperm capacitation (Murdica *et al.* 2019*b*, Wang *et al.* 2022) and the acrosome reaction (Murdica *et al.* 2019*b*, Palmerini *et al.* 2003). However, our findings do align with studies demonstrating only subtle impacts on sperm motility following sperm-SFEV interactions unless the sperm response to SFEVs is augmented by the inclusion of additional signalling mediators (Arienti *et al.* 2001, Palmerini *et al.* 2003, Park *et al.* 2011). Additionally, these data align with studies showing that SFEVs either failed to elicit any effect on, or inhibited, sperm capacitation and the induction of an acrosome reaction (Bechoua *et al.* 2011, Pons-Rejraji *et al.* 2011). Thus, while SFEVs may deliver functional mediators to sperm at ejaculation, the full functional consequences of these proteins may only be apparent when the spermatozoon encounters specific physiological environments within the uterus and oviduct.

It is important to note that there is interest in utilising SFEVs in the diagnosis and treatment of infertility in human subjects. Indeed, SFEV cargo is dysregulated in asthenozoospermic (decreased percentage of progressively motile sperm) males compared to normozoospermic males (Lin *et al.* 2019, Murdica *et al.* 2019*a*,*b*). Among the dysregulated cargo, the aforementioned CRISP1, glycodelin, and TRPV6 proteins (Lin *et al.* 2019, Murdica *et al.* 2019*a*,*b*) could conceivably influence sperm function in these individuals. To address SFEV-driven sperm defects, some studies have proposed using strategies such as the supplementation of normozoospermic SFEVs to asthenozoospermic donor samples to improve sperm motility (Goss *et al.* 2023). Alternatively, studies in animal models have suggested that the supplementation of mesenchymal-derived stem cell EVs can be used to improve sperm quality parameters following cryopreservation (Mokarizadeh *et al.* 2013, Qamar *et al.* 2019). However, our results encourage caution to ensure that sperm-SFEV interactions occur in an environment that allows these cells to realise their fertilising potential.

Collectively, these data demonstrate subtle contributions of SFEVs toward the regulation of human sperm function. However, an important caveat to the interpretation of these data is that the human sperm samples used in these studies were isolated from ejaculated samples that had completed an extended period of incubation in the presence of seminal fluid, as is common practice in andrological studies. While such an approach is permissive of liquefaction and has been used in studies that have shown an influence of SFEVs over sperm function (Palmerini *et al.* 1999, Palmerini *et al.* 2003, Murdica *et al.* 2019*b*, Wang *et al.* 2022), this practice also raises the prospect that the recipient sperm populations may have inadvertently become saturated with SFEVs and thus refractory to their functions. To address this, studies have collected populations of spermatozoa (Park *et al.* 2011) prior to seminal fluid liquefaction, but whether such collection could occur rapidly enough to prevent endogenous sperm–SFEV interactions remains unclear. Given the rapid nature of sperm–SFEV interactions (within 1 min), it appears likely that no isolation approach will completely avoid endogenous interactions. Indeed, we attempted to isolate sperm as rapidly as possible following donation, but even under ideal conditions, as much as 15 min elapse prior to the recovery of a usable sperm population, and these sperm again show minimal responsiveness to co-culture with SFEVs.

In summary, herein we report the enrichment of human SFEVs using protocols developed in accordance with MISEV guidelines (Thery *et al.* 2018). We also demonstrated that SFEVs formed productive interactions with homologous spermatozoa, delivering protein cargo that appeared to distribute throughout the entire sperm cell. Despite the potential implications of such exchange, SFEV co-incubation had only subtle effects on the functional profile of spermatozoa. There are several caveats that must be considered in the interpretation of these data, including the influence of endogenous SFEVs that may negate responses to additional SFEV cargo. Nevertheless, such findings raise the prospect that mature spermatozoa may not be the sole/primary target for SFEVs and that their encapsulated cargo may also regulate other physiological features of the reproductive process, such as conditioning the female reproductive tract environment to prepare for pregnancy (Schjenken & Robertson 2020, Tamessar *et al.* 2021).

## Supplementary Materials

Supplementary Table 1. Details of antibodies used throughout this study

Supplementary Figure 1: Viability of spermatozoa incubated at pH 5 and pH 7.

Supplemental Figure 2: Overall spermatozoa motility is not influenced by seminal fluid extracellular vesicles incubation in an acidic (pH 5) environment.

Supplemental Figure 3: Spermatozoa motility is not influenced by seminal fluid extracellular vesicles incubation in a neutral pH (pH 7) environment.

Supplemental Figure 4: Seminal fluid extracellular vesicles do not influence motility parameters of high-quality sperm collected prior to liquification.

Supplemental Figure 5: Seminal fluid extracellular vesicles do not influence motility parameters of high-quality sperm collected prior to liquification.

## Declaration of interest

BN is a member of the Editorial Board of *Reproduction & Fertility*but was not involved in the peer review process of this paper. All other authors declare no conflicts of interest that could be perceived as prejudicing the impartiality of the research reported.

## Funding

This work was supported by the National Health and Medical Research Councilhttp://dx.doi.org/10.13039/501100000265 of Australia (NHMRC; grant number APP1147932, 2018-2020 and APP2019934, 2023–2026). BN is the recipient of an NHMRC Senior Research Fellowship (grant number APP1154837, 2019–2023). EGB is the recipient of an Australian Research Councilhttp://dx.doi.org/10.13039/501100000923 Discovery Early Career Researcher Fellowship (grant no. DE210100103 2022–2024). CT is the recipient of a University of Newcastle Australian Governmenthttp://dx.doi.org/10.13039/100015539 Research Training Program postgraduate research scholarship.

## Author contribution statement

CT performed experiments, analysed and interpreted data, and drafted the manuscript. ALA, NAT, SP, and SJS performed experiments and analysed data. EGB supervised CT and contributed to data interpretation. JW, HMZ, SAR, and DJS contributed to data interpretation. BN and JES conceived the study, supervised CT, and analysed and interpreted data. All authors read, edited, and approved the manuscript.
