# Supplementary material for: The efficacy and functional consequences of interactions between human spermatozoa and seminal fluid extracellular vesicles
Source: Reprod Fertil. 2024 Oct 4;5(4):e230088. doi: 10.1530/RAF-23-0088 (PMC11466262; doi:10.1530/RAF-23-0088)
Supplement: Supplementary Table 1. Details of antibodies used throughout this study [file supplementary_table_1.pdf]

Supplementary Table 1. Details of antibodies used throughout this study

Antibody	Final concentration (dilution of stock solution)*		Company	Catalogue N$^{o.}$	Batch N$^{o.}$	Stock Concentration
Primary antibodies	**IF**	**IB**				
Flotillin 1 (anti-FLOT1)	-	0.1 µg (1:10000)	Sigma Aldrich	F1180	128M4839V	1 mg/ml
Apolipoprotein A1 (anti-APOA1)	-	3.0 µg (1:333)	Abcam	ab33470	GR3256297-8	1 mg/ml
anti-CD63	-	1.0 µg (1:1000)	GeneTex	GTX17441	822002616	1 mg/ml
Anti-Phosphotyrosine, Mouse monoclonal (PT66)	8 µg (1:250)	-	Sigma Aldrich	P5872	127M470V	2 mg/ml
Lectin PNA From Arachis hypogaea (peanut), Alexa Fluor 594 Conjugate	5 µg (1:200)	-	ThermoFisher Scientific	L32459	1715258	1 mg/ml
Secondary antibodies						
Goat anti rabbit HRP	-	0.05 µg (1:2500)	Merck	DC03L	N/A	0.13 mg/ml

Streptavidin Alexa Fluor 488 conjugate	5.0 µg (1:400)	-	Thermo	S11223	2277786	2 mg/ml
Goat anti-Mouse IgG (H+L), Alexa Fluor 488	1.5 µg (1:400)	-	Thermo	A-11001	237967	1 mg/ml

*IF, immunofluorescence; IB, immunoblot; -, not applicable; N/A, information not available from manufacturer
