# Supplementary figures and images for: The efficacy and functional consequences of interactions between human spermatozoa and seminal fluid extracellular vesicles
Source: Reprod Fertil. 2024 Oct 4;5(4):e230088. doi: 10.1530/RAF-23-0088 (PMC11466262; doi:10.1530/RAF-23-0088)

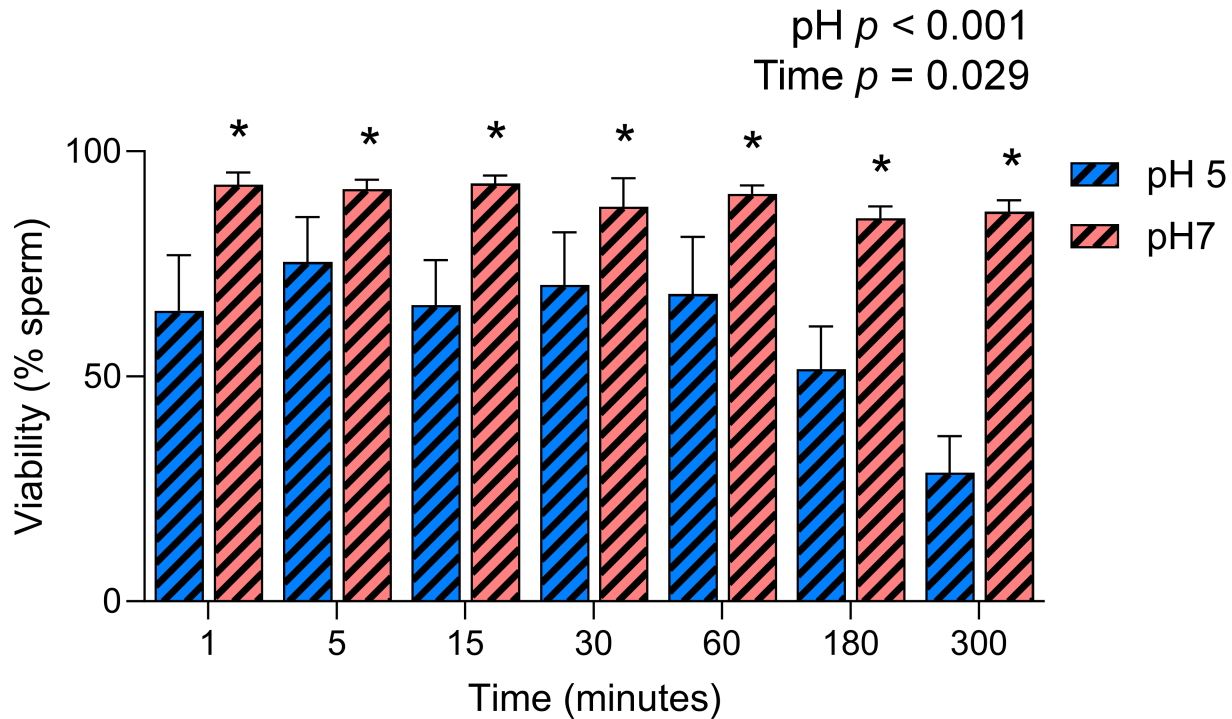

Supplement: Supplementary Figure 1: Viability of spermatozoa incubated at pH 5 and pH 7. [file supplementary_figure_1.pdf]

# High quality spermatozoa isolation prior to liquification and incubated at pH 5

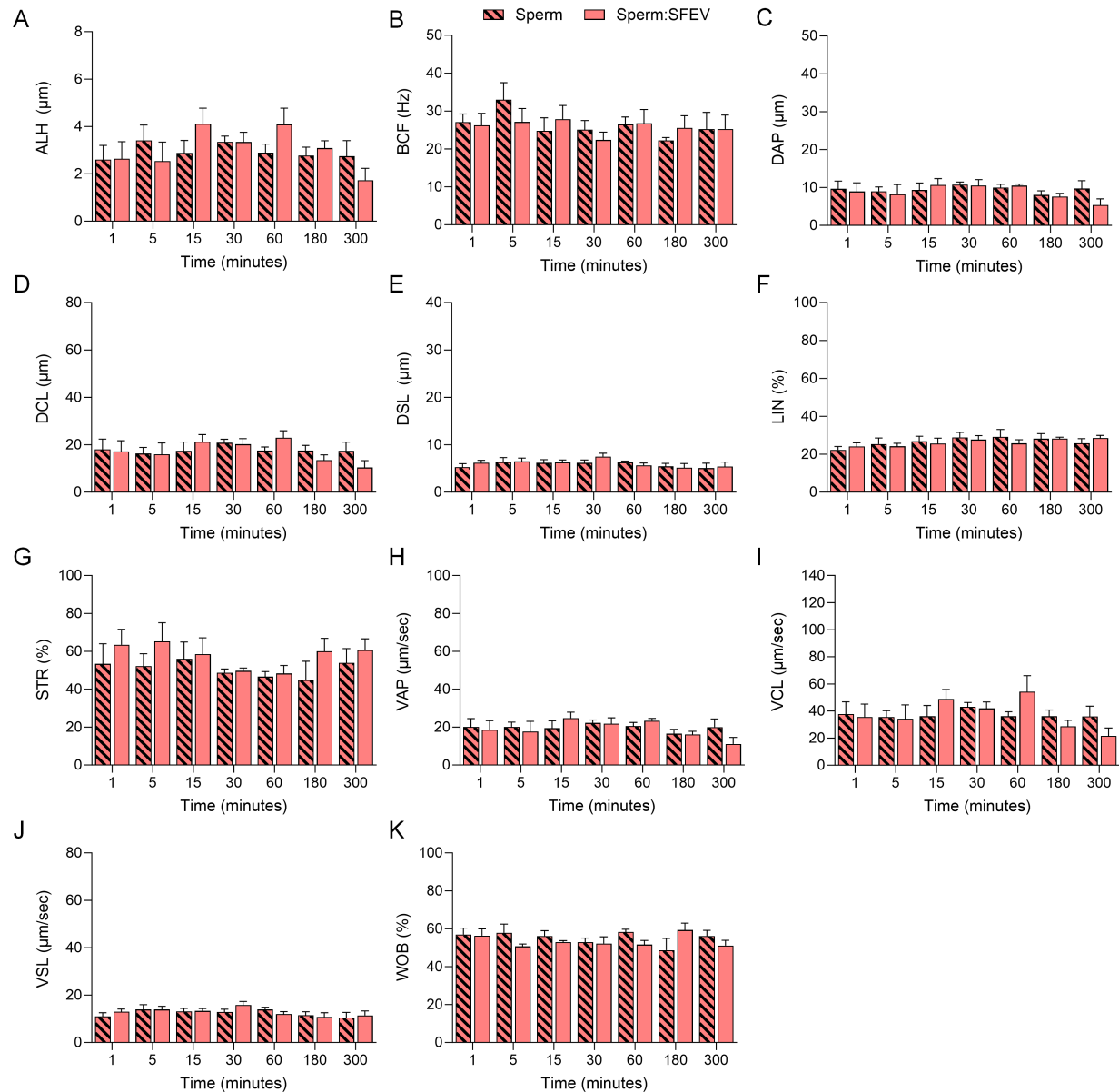

Supplement: Supplemental Figure 2: Overall spermatozoa motility is not influenced by seminal fluid extracellular vesicles incubation in an acidic (pH 5) environment. [file supplementary_figure_2.pdf]

# High quality spermatozoa isolation prior to liquification

■ Sperm ■ Sperm:SFEV

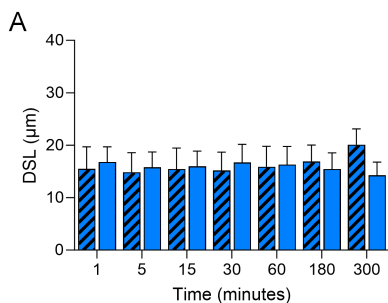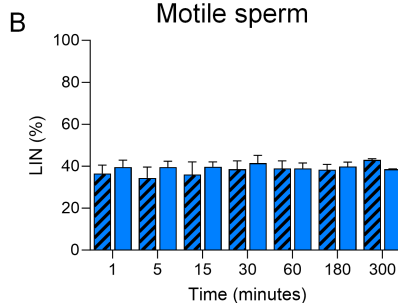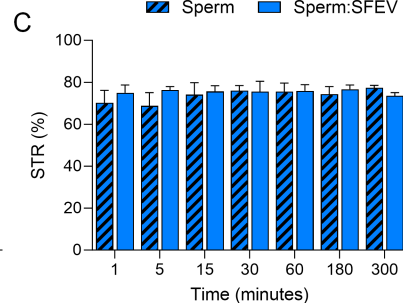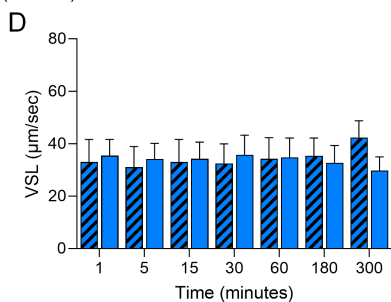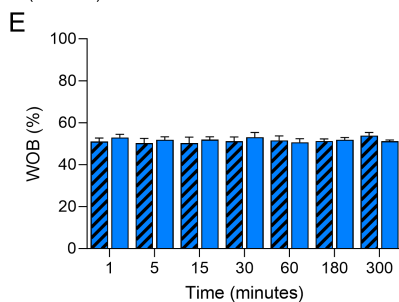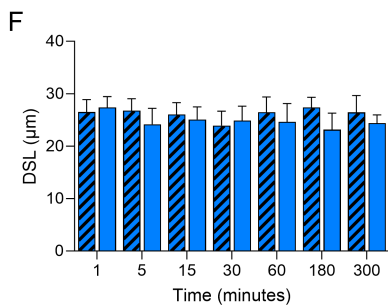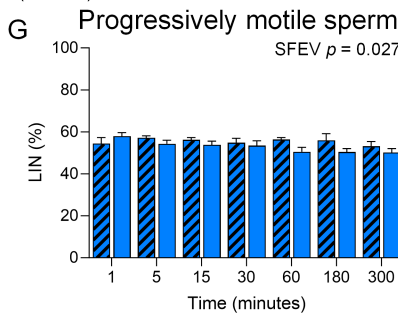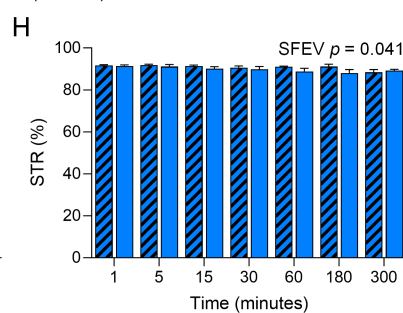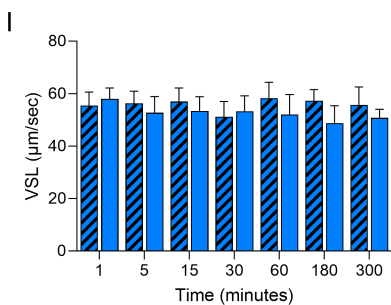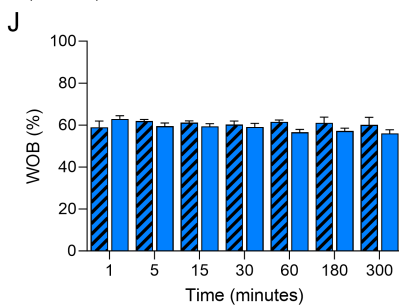

Supplement: Supplemental Figure 4: Seminal fluid extracellular vesicles do not influence motility parameters of high-quality sperm collected prior to liquification. [file supplementary_figure_4.pdf]

# Low quality spermatozoa isolation prior to liquefaction

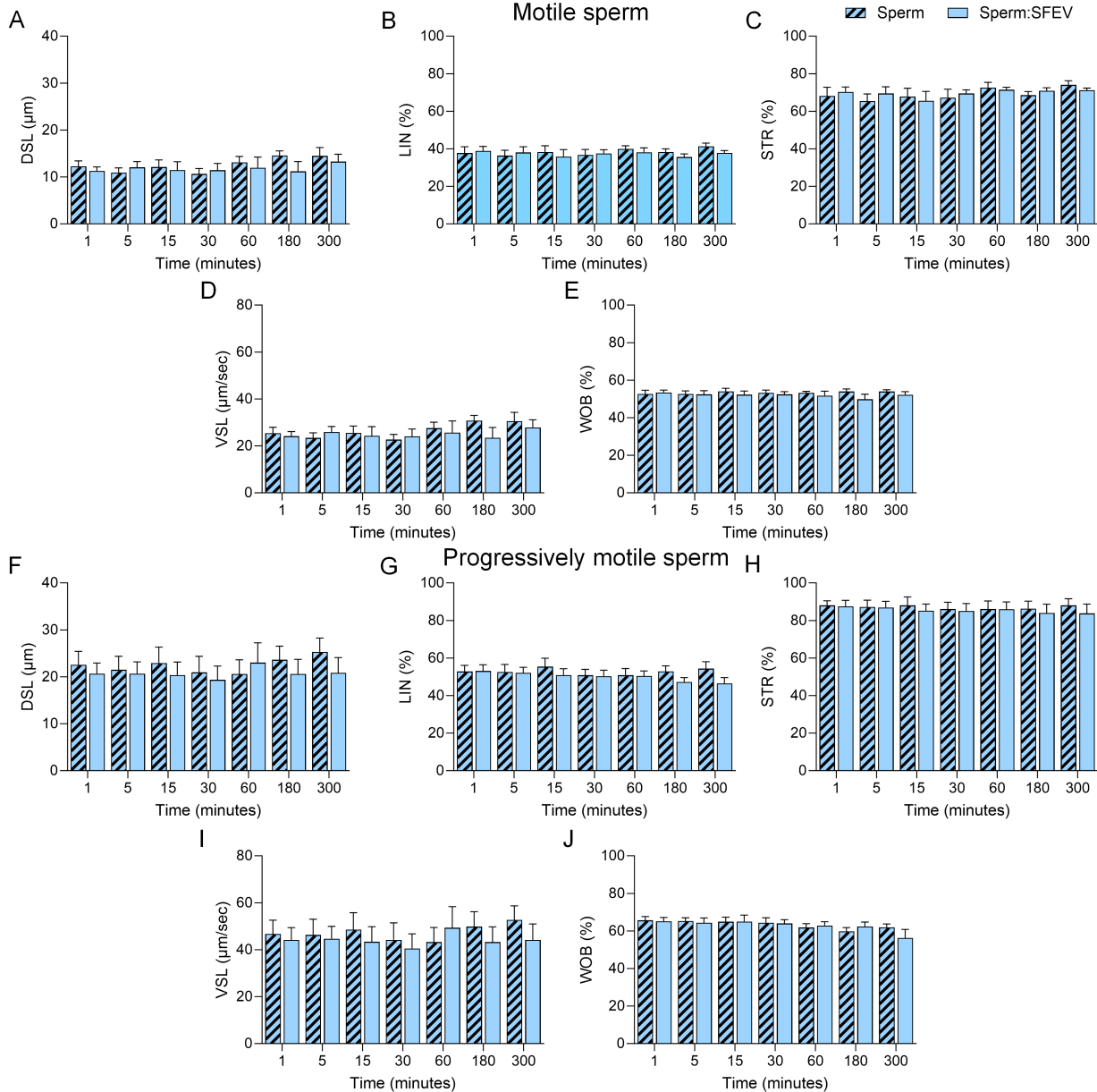

Supplement: Supplemental Figure 5: Seminal fluid extracellular vesicles do not influence motility parameters of high-quality sperm collected prior to liquification. [file supplementary_figure_5.pdf]
