# Supplementary material for: The efficacy and functional consequences of interactions between human spermatozoa and seminal fluid extracellular vesicles
Source: Reprod Fertil. 2024 Oct 4;5(4):e230088. doi: 10.1530/RAF-23-0088 (PMC11466262; doi:10.1530/RAF-23-0088)
Supplement: Supplemental Figure 3: Spermatozoa motility is not influenced by seminal fluid extracellular vesicles incubation in a neutral pH (pH 7) environment. [file supplementary_figure_3.pdf]

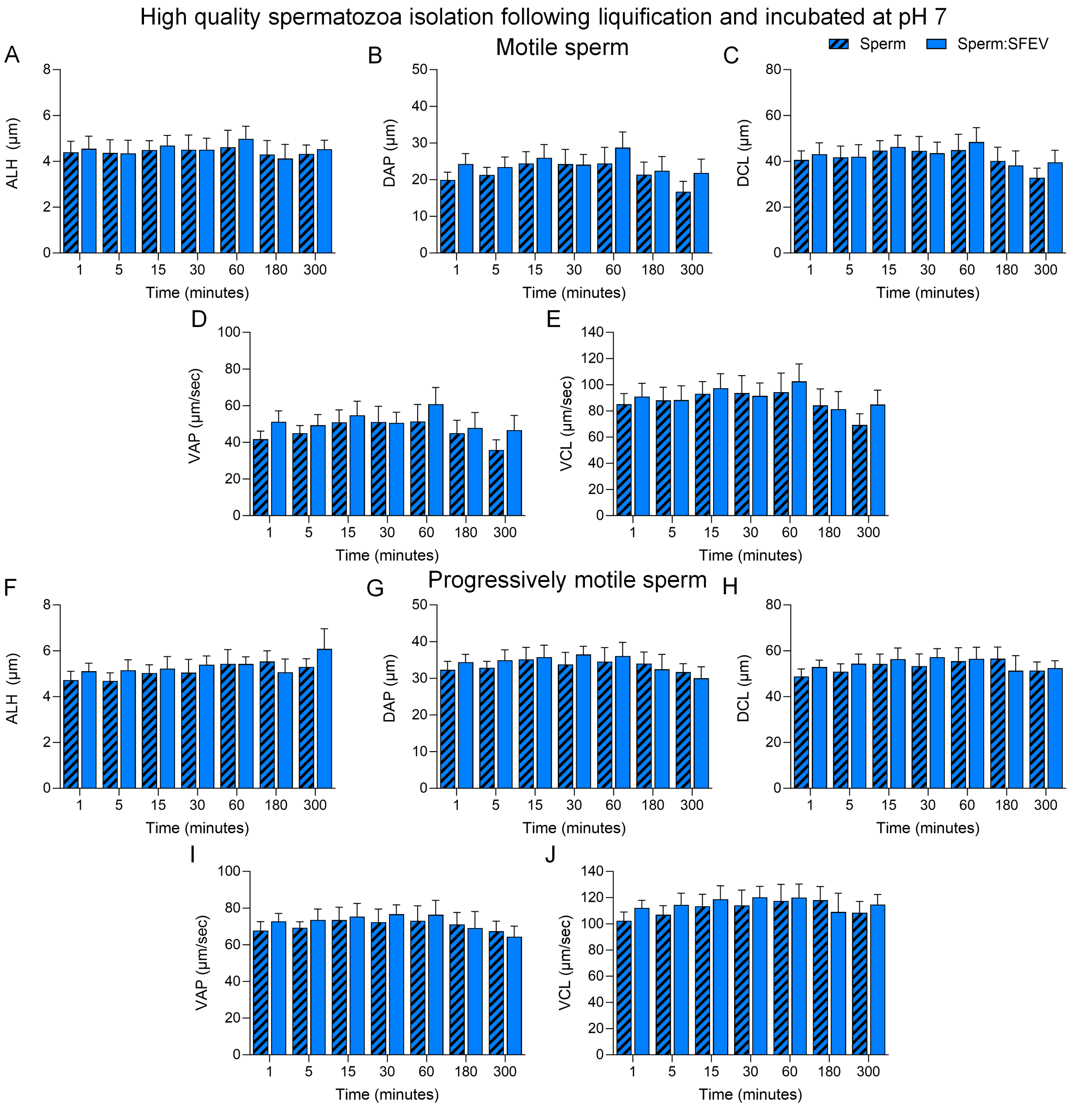
High quality spermatozoa isolation following liquification and incubated at pH 7
Motile sperm
Sperm
Sperm:SFEV
A
B
C
D
E
ALH (µm)
DAP (µm)
DCL (µm)
VAP (µm/sec)
VCL (µm/sec)
Time (minutes)
1
5
15
30
60
180
300
Progressively motile sperm
F
G
H
I
J
